# Organic radicals stabilization above 300 °C in Eu-based coordination polymers for solar steam generation

**DOI:** 10.1038/s41467-022-33948-9

**Published:** 2022-10-17

**Authors:** Xinhe Ye, Lai-Hon Chung, Kedi Li, Saili Zheng, Yan-Lung Wong, Zihao Feng, Yonghe He, Dandan Chu, Zhengtao Xu, Lin Yu, Jun He

**Affiliations:** 1 School of Chemical Engineering and Light Industry, Guangdong University of Technology, Guangzhou, 510006 China; 2 Department of Chemistry, City University of Hong Kong, 83 Tat Chee Avenue, Kowloon, Hong Kong, China; 3 Institute of Materials Research and Engineering (IMRE), Agency of Science, Technology and Research (A*STAR), 2 Fusionopolis Way, Singapore, 138634 Republic of Singapore

**Keywords:** Inorganic chemistry, Coordination chemistry, Materials for energy and catalysis, Metal-organic frameworks

## Abstract

Organic radicals feature unpaired electrons, and these compounds may have applications in biomedical technology and as materials for solar energy conversion. However, unpaired electrons tend to pair up (to form chemical bonds), making radicals unstable and hampering their applications. Here we report an organic radical system that is stable even at 350 °C, surpassing the upper temperature limit (200 °C) observed for other organic radicals. The system reported herein features a sulfur-rich organic linker that facilitates the formation of the radical centers; on the solid-state level, the molecules are crystallized with Eu(III) ions to form a 3D framework featuring stacks of linker molecules. The stacking is, however, somewhat loose and allows the molecules to wiggle and transform into sulfur-stabilized radicals at higher temperatures. In addition, the resulting solid framework remains crystalline, and it is stable to water and air. Moreover, it is black and features strong broad absorption in the visible and near IR region, thereby enhancing both photothermal conversion and solar-driven water evaporation.

## Abstract (web summary)

Organic radicals have potential applications in a variety of fields, including energy conversion. Here, the authors report Eu-based coordination polymers that enable the stabilization of organic radicals up to 350 °C; these systems can be used to enhance solar steam generation.

## Introduction

Organic radicals are often reactive and unstable species, because their unpaired electrons tend to pair to form covalent bonding, and for achieving the stable doubly occupied (closed shell) configuration. Stabilized radicals^1,2^, on the other hand, allow for many uses in polymer functionalization^3–5^, solar cells^6,7^, magnetic materials design^8–11^, and bioimaging/cancer therapy^12,13^. To enhance stability, bulky groups can be piled around the radical center to afford steric protection^2,14,15^ and large conjugate molecules can be used to host the radical center, so as to delocalize the unpaired electron and to mitigate its open-shell character^16–21^. Both are known molecular design strategies that can yield refreshing results, like the dicarbonyl radical cation persistent even at 200 °C^14^, and 200 °C is indeed the upper limit achieved so far for radicals.

In the solid-state approach, the tailorable spatial order of framework materials offers an opportunity to trap reactive species and to channel reaction pathways^22–30^. Notably, Matsuda and Ma reported spatially isolated carbene sites at the midpoints of the long linkers in a rigid open metal-organic framework (MOF)^31^, and their higher decomposing temperature (170 K), relative to the control carbene species (80 K; in a frozen matrix of 2-methyltetrahydrofuran). Also, similarly anchored -SI and -SH groups on porous MOF grids had been explored to prevent, respectively, disproportionation (into -SS- and I_2_)^32^, and poisoning of the Pd centers of the leach-free heterogeneous MOF catalyst^33^. In these very open nets, the far-apart, isolated linkers experienced lesser steric interaction from one another, and the reactivity control by molecular packing (e.g., topochemistry) was therefore weakened.

**Fig. 1:**
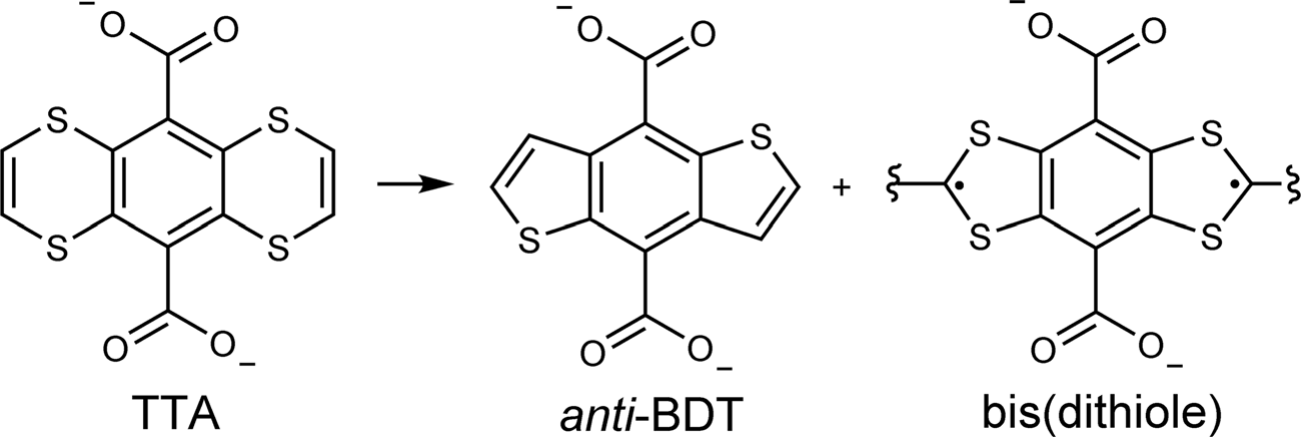
Transformation of the TTA linker in the EuTTA crystal. The chemical structures of the TTA linker molecule (left), the *anti*-BDT (middle), and bis(dithiole) (right) species; the latter two are generated by heating the EuTTA crystal.

In this work, we report an Eu-based MOF scaffold that enables the generation of organic radical species stable even at 350 °C. The approach followed herein synergizes molecular design and spatial control in the solid state. Specifically, we build the potentially reactive 1,4-dithiin functions into the linker molecule (Fig. 1), which then, in the resultant MOF scaffold of EuTTA, are arranged as to be slightly spaced apart (e.g., by *ca*. 1.0 Å above VDW contact). The loose packing allows for some motion, enabling reactions to occur, while the spatial restriction narrows down the reaction pathways to better target the organic radical products.

## Results and discussion

### Framework formation and crystal structure

The framework builds on the molecule H_2_TTA (1,4,5,8-tetrathiaanthracene-9,10-dicarboxylic acid, Fig. 1), which is equipped with the carboxylic groups widely used for MOF construction^34–37^. But the key design lies in the two wings of 1,4-dithiin sulfur units, as these 8π-electron, antiaromatics offer rich reactivities. For example, in various reactions on the ethene and S functions, ring contraction can occur to form the 6e^−^ aromatic thiophenes^38,39^; moreover, the alternative, 1,3-dithiole radical^40,41^ product (Fig. 1) has not been generated from dithiin precursors, but would enrich radical-containing MOF solids^42–57^.

**Fig. 2:**
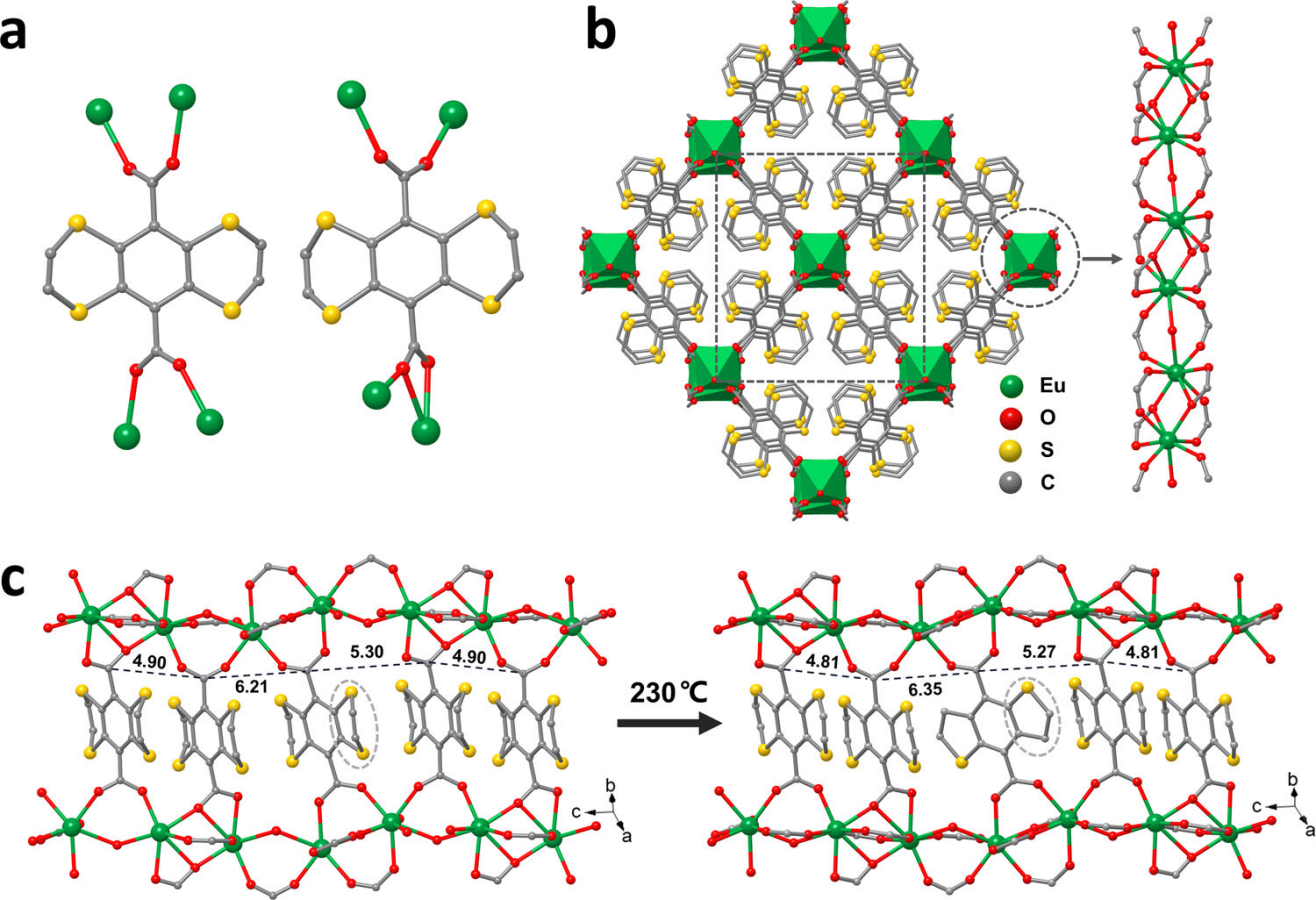
Single-crystal-to-single-crystal change from EuTTA to EuTTA-230. **a** Diagrammatic representation of the coordination environment of two types of coordination modes between carboxylate and Eu centers. **b** A view of EuTTA along the *c* axis. **c** The side view of a stack of the linker molecules and the two associated Eu-carboxylate chains in EuTTA and EuTTA-230. The numbers (in Å) mark the spacing between the neighboring carboxyl C atoms. Note: the thiophene portion in panel **c** is disordered and can also be refined as a dithiole unit.

**Fig. 3:**
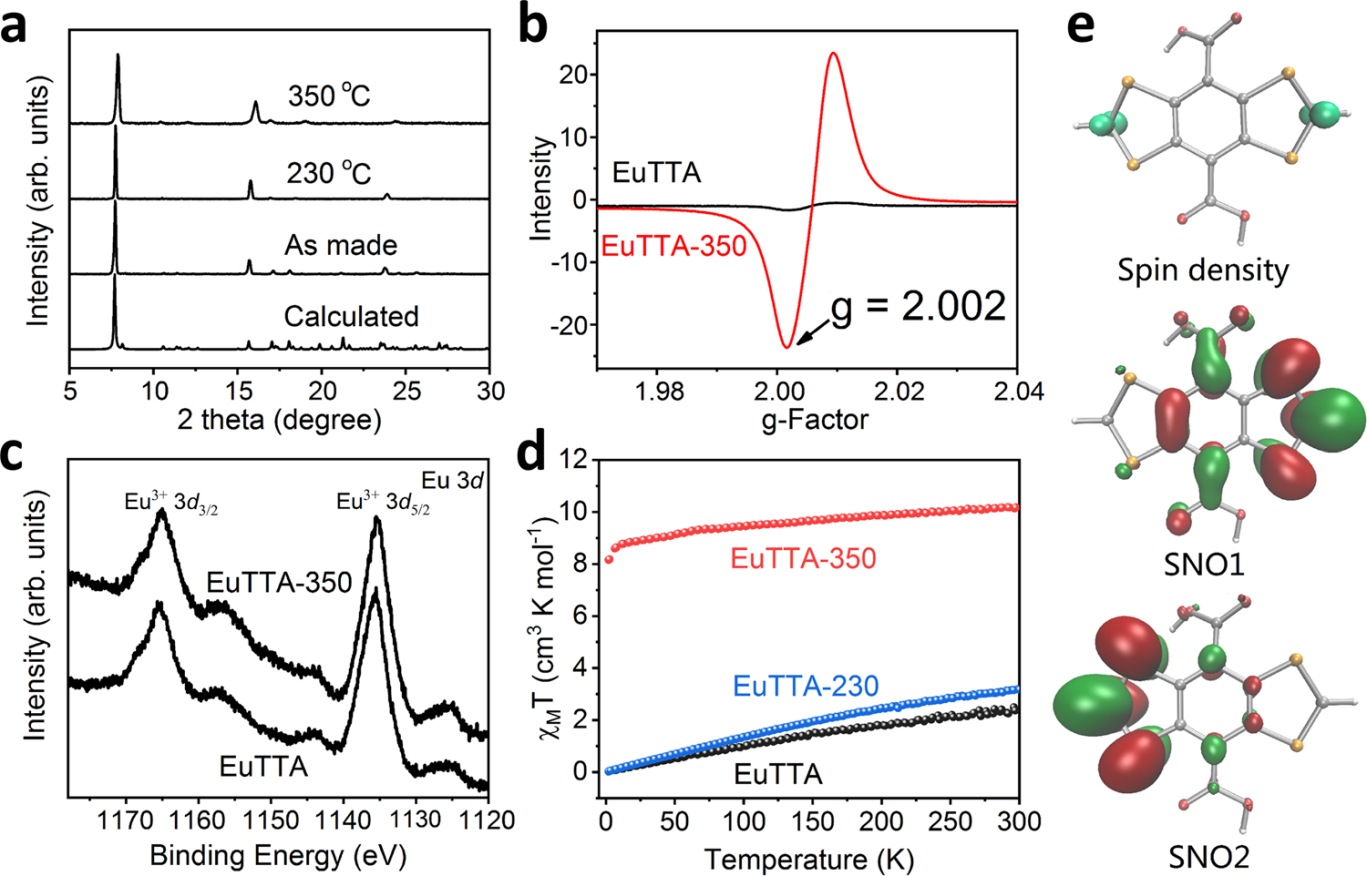
Characterization of the crystalline solids of EuTTA and its radical-containing derivatives. **a** Powder X-ray diffraction patterns of EuTTA, EuTTA-230, and EuTTA-350. **b** Solid-state EPR spectra of EuTTA and EuTTA-350 at 300 K. **c** The XPS spectra of Eu 3*d* of EuTTA and EuTTA-350. **d** Plots of ${{\chi }}_{{{{{{\boldsymbol{M}}}}}}}{{{{{\boldsymbol{T}}}}}}$ versus T for EuTTA, EuTTA-230, and EuTTA-350 at 1000 Oe field. **e** Spin densities distribution of the triplet biradical bis(dithiole) with an isosurface value of 0.02 au. and calculated SNOs of independent spins (red area stands for negative value and green area for positive value).

In practice, single crystals of EuTTA were obtained by reacting H_2_TTA with EuCl_3_ ∙ 6H_2_O in water and acetonitrile (4:1, v/v) at 150 °C for 48 h. The yellow crystals are not photoluminescent^58^. The X-ray structure of the 3D net of EuTTA features parallel Eu-carboxyl rods (along the *c* axis) in a quadrangle array (Fig. 2). The neighboring Eu atoms exhibit alternating distance (3.948 and 4.517 Å): the short pair is straddled by four carboxyl groups, and the long pair by two carboxyl groups and one aqua bridge. Together the asymmetric formula of the unit cell is Eu(C_12_H_4_O_4_S_4_)_1.5_(H_2_O)_0.5_, with one centric TTA piece (with lower site multiplicity) contributing only as half. The bulk sample features the same crystalline phase (PXRD patterns in Fig. 3a). Elemental analysis found [C (31.65 %), H (1.24 %), S (24.62 %)], fitting the formula Eu_2_(C_12_H_4_O_4_S_4_)_3_(H_2_O)_2_ with a calculated profile: C (31.77 %), H (1.18 %), S (28.26%).

The spacing of the TTA molecules along the Eu-carboxyl rod is also uneven (Fig. 2c): with a close pair (interplanar gap: 3.7 Å; the pair is related by a center of symmetry, but each being non-centric) alternating with the (centric) TTA at *ca*. 4.6 Å. The C_2_H_2_ flank of the latter is disordered over two sets of positions, in line with the more open setting. The opening, however, is limited: it allows the atoms to wiggle more, but not N_2_ or CO_2_ guests to sorb (Supplementary Fig. 25).

The impact on reactivity, notably, is decisive. For when the EuTTA crystal is heated to 230 °C (to form EuTTA-230), the loose (centric) TTA loses one (S or C) atom on each wing, and forms benzodithiophene (BDT) or bis(dithiole) rings (as illustrated in Figs. 1 and 2c); the other two (i.e., the close pair of) TTA, by comparison, remain unchanged. As evidence, the X-ray structure is telling (Fig. 2c). The heated sample (EuTTA-230) retains the same net and the same space group [C2/c: 16.385(3), 16.484(3), 16.295(3) Å, 116.11(3)°; cell volume: 3952.0 Å^3^], *cf*. the pristine EuTTA [16.5676(15), 16.3937(14), 16.2839(14) Å; cell volume: 4045.5 Å^3^]. Revealingly, the sulfur atoms on the transformed linker become only partially occupied (i.e., contrasting the full occupancy of the dithiin S sites in the other two unchanged TTA pieces). The disordering of the S and C sites on the transformed linker, however, precludes a qualitative assignment of the benzodithiophene (BDT) or bis(dithiole) components. But the presence of the reported *anti*-benzo[1,2-b:5,4-b’]dithiophene molecule (*anti*-BDT; see SI for our modified synthetic procedure and Supplementary Figs. 13–18 for the NMR and MS data)^59^ is supported by solution NMR data (from the EuTTA-230 sample dissolved in HF/DMSO-*d*_*6*_; mostly dissolved, but with a little black solid remaining), see Supplementary Fig. 27, while the distinct EPR signal of the EuTTA-230 solid (Supplementary Fig. 28) is consistent with the formation of the open-shell dithiole species. Besides the *anti*-BDT signals, the NMR data (Supplementary Fig. 27) also feature additional peaks, suggesting other molecular products in the bulk sample, including possibly the *syn*-BDT isomer and the halfway, dithiin-benzothiophene product DTBT as drawn in Supplementary Fig. 27.

The ring contraction lessens the steric repulsion on the carboxyl groups, and more coplanarity is seen between the benzothiophene and carboxyl moieties, with the dihedral angle (5.16°) much smaller than that of the dithiin-based TTA precursor (48.65°). The coplanarity helps align the benzothiophene length along the Eu-carboxyl rod, and swings its wing atoms closer to the neighboring dithiin molecules (at C ∙ ∙ ∙ C contact of 3.49 Å). The wiggle room for the molecules is, therefore, compressed, apparently stopping the two remaining TTA linkers from undergoing reactions at this temperature (230 °C).

At higher temperatures (e.g., 350 °C for 2 h), the remaining TTA linkers also react. Specifically, the resulting crystals (EuTTA-350) become darker (black; Supplementary Fig. 24) and slower to dissolve in HF/DMSO-*d*_*6*_, and with a little more black solid remaining relative to the EuTTA-230 case (see Supplementary Figs. 36–39 for the PXRD, IR and EDX characterization of the residue). The dissolved portion features only two pairs of peaks of ^1^H NMR (Supplementary Fig. 27): one from the known *anti*-BDT, the other possibly from the *syn*-BDT isomer), without any dithiin C_2_H_2_ signal from the TTA molecule remaining. On the other hand, attention to the black solid residue, albeit insoluble and amorphous, is deserving. X-ray absorption fine structure (XAFS) techniques, for example, could unveil the coordination environment of the Eu atom therein, so as to help explain its resistance against being extracted by DMSO/HF. These and other characterizations are better left to future studies, where larger scales of preparation would also accumulate a greater quantity of this residue for investigation.

### Radical analysis of EuTTA-350

**Fig. 4:**
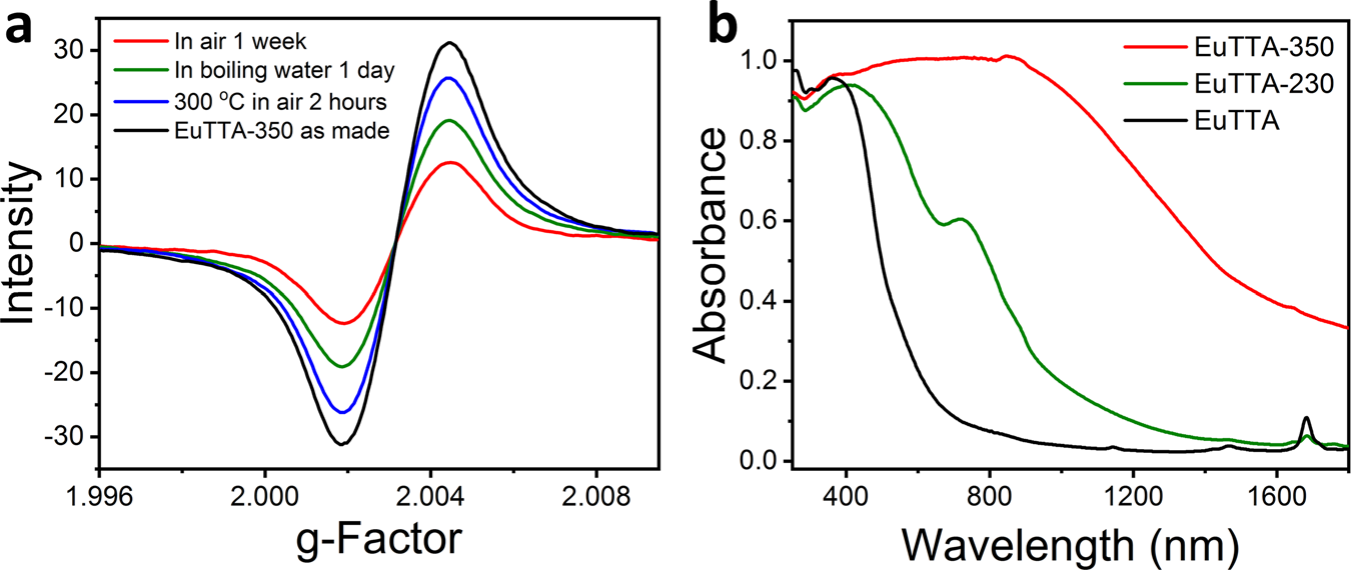
Characterization of the stability and light absorption of EuTTA-350. **a** The solid-state electron paramagnetic resonance (EPR) spectrum of EuTTA-350 treated under different conditions. **b** UV-Vis-NIR absorption of EuTTA, EuTTA-230, and EuTTA-350 powders.

Most notable of the EuTTA-350 crystals is the stronger and stable paramagnetic signals. The EPR signal centers at *g* = 2.002 (Fig. 3b), and is indicative of organic radicals. No EPR peaks for Eu(II) (e.g., *g* ≈ 2.0, 2.8, 3.4, 4.5, and 6.0)^60^ were detected, with the 4*f*^6^ Eu^3+^ (in the ^7^F_0_ ground state) being diamagnetic/EPR-silent. No reduction of Eu(III) to Eu(II) during thermal treatment was thus found, which is also confirmed by the X-ray photoelectron spectroscopy (XPS) signals at 1165.5 and 1135.4 eV corresponding to the Eu(III) 3*d*_3/2_ and 3*d*_5/2_ peaks which are also observed in the EuTTA precursor (Fig. 3c and Supplementary Figs. 30 and 31)^61,62^. The EPR signal of EuTTA-350 remains significant after heating at 300 °C in the air for 2 h, or boiling in water for 24 h (Fig. 4a). Notably, the organic components of EuTTA-350 can be extracted into an acidic solution (e.g., into DMSO/HCl), and then precipitated (e.g., by adding water) to give an organic solid that retains strong EPR signal, further showcasing the stability of the radical species thereof (Supplementary Fig. 29).

The stronger EPR signal suggests the further formation of 1,3-dithiole-based radicals in EuTTA-350, i.e., from the two remaining TTA linkers, in addition to the radical species, initially formed (e.g., at 230 °C, as in the above mentioned EuTTA-230). The strong paramagnetism complicates the direct detection of the dithiole products by NMR. To verify the formation of a bis(dithiole) molecule, it is key to demonstrate the retention of the four sulfur atoms (vs. only two S atoms in the benzodithiophene case). For this, oxidative treatment to convert the bis(dithiole) into the more tractable tetrasulfonic acid derivative (Supplementary Fig. 33)^63^ was attempted. Specifically, the soluble fraction of the EuTTA-350 crystals were first extracted into DMSO/HCl, and then precipitated out by adding water. The black precipitate was treated with 30% H_2_O_2_ to give a red-brown solution (with some red solid remaining); the solution was then evaporated, and the residue (dissolved in D_2_O) was found by both ^1^H and ^13^C NMR to feature the expected tetrasulfonic acid derivative (Supplementary Fig. 33).

Single-crystal X-ray diffraction of EuTTA-350 remains strong (albeit with peaks of poor shapes), and indicates the structural integrity of the Eu-carboxylate framework. But the severe disorder on the linker portion makes it hard to pinpoint the C and S atoms on the two S-heterocycle wings (see the.res file for a plausible but non-definitive model for the flanking S/C sites). So the X-ray data of EuTTA-350 do not clarify the crosslinks among the linker molecules; but the lowered solubility of the EuTTA-350 crystals in HF/DMSO-*d*_6_ (relative to the readily soluble EuTTA), as well as the high molecular weights (average *ca*. 9000) determined by gel permeation chromatography (GPC; Supplementary Fig. 34), indicate substantial crosslinking, as is tentatively proposed in Supplementary Fig. 40; the scheme therein features the formula Eu_4_(C_24_H_6_O_8_S_4_)(C_23_H_6_O_8_S_8_)_2_(H_2_O)_14_, and a calculated elemental profile [C (30.64 %), H (1.69 %), S (23.37 %)] consistent with the elemental analysis results [C (30.71 %), H (1.85 %), S (25.02 %] for EuTTA-350. The loss of carbon atoms, as suggested in Supplementary Fig. 40, is also supported by the TG-GC-MS monitoring of the gas release from the heated EuTTA sample (Supplementary Fig. 35); specifically, a distinct mass-to-charge peak of 28.1 was detected, and indicated the emission of ethylene (with N_2_ being excluded because the carrier gas is Ar).

Variable-temperature magnetic susceptibility measurements of EuTTA and EuTTA-350 were carried out at a dc field of 1000 Oe at 2‒300 K. From ${{\chi }}_{M}T$ versus *T* plots (Fig. 3d), ${{\chi }}_{M}T$ of EuTTA is 2.38 cm^3^ K mol^−1^ at room temperature and decreased to 0.025 cm^3^ K mol^−1^ at 2 K. The paramagnetism of EuTTA in 2‒300 K is caused by coupling between nonmagnetic ^7^F_0_ ground state and closely lying ^7^F_1_ excited state of Eu(III)^64^, so the ${{\chi }}_{M}T$ contributed by Eu(III) is 2.36 cm^3^ K mol^−1^. As for EuTTA-350 [molar mass 1209: 90% of that of EuTTA, Eu_2_(C_12_H_4_O_4_S_4_)_3_(H_2_O), 1343; based on TGA weight loss of 10% from EuTTA to EuTTA-350, Supplementary Fig. 22], the ${{\chi }}_{M}T$ is 10.06 cm^3^ K mol^−1^ at room temperature and decreased to 8.09 cm^3^ K mol^−1^. The decrease is caused by Eu(III), while the remaining ${{\chi }}_{M}T$ value of 8.09 cm^3^ K mol^−1^ can be ascribed to the organic radicals in EuTTA-350. The corresponding effective magnetic moment μ_eff_ = (8${{\chi }}_{M}T$)^1/2^ = 8.04 B.M., which approximates that of six unpaired electrons: μ_eff_ = $g\sqrt{S(S+1)}$ = 6.93 (S = 6/2 = 3, with *g* = 2). Because of little linker loss (e.g., <5% from TGA data, Supplementary Fig. 22) from EuTTA to EuTTA-350, we assume the formula unit of EuTTA-350 to retain three linkers; the above numbers thus suggest each linker bearing two unpaired electrons as a diradical. The biradical is fitting for the bis(dithiole) product and was also consistent with the tetrasulfonic derivative (Supplementary Fig. 33); As for the benzodithiophene (BDT) components, radicals can arise and stabilize from thiophene crosslinks as illustrated in Supplementary Fig. 40^6^.

### DFT calculation

For illustration, a theoretical calculation was conducted on the protonated form of the bis(dithiole) diradical (Fig. 3e). The results show that spin densities locate mainly on the two sulfur-flanked C atoms (0.71 and 0.63 spin, respectively, Supplementary Table 1); the S atoms also take up almost the remaining spin densities (0.28 and 0.17 spin on each set of S atoms, Supplementary Table 1). Atoms of the benzenoid core also share spin densities, but these are far smaller than the spin densities on the side S‒C‒S moieties. The spin natural orbitals (SNOs) of two independent spins (Fig. 3e) suggest that (1) two spins mainly localize on the S‒C‒S wings (71.72 and 88.48% orbital contribution, Supplementary Table 2) and (2) the two spins are unlike to mix as reflected by the separate orbitals occupied by each spin. The spin densities and SNO results thus consistently indicate the diradicals to mostly localize on the S‒C‒S units of the dithiole rings.

### Photothermal conversion and water evaporation

**Fig. 5:**
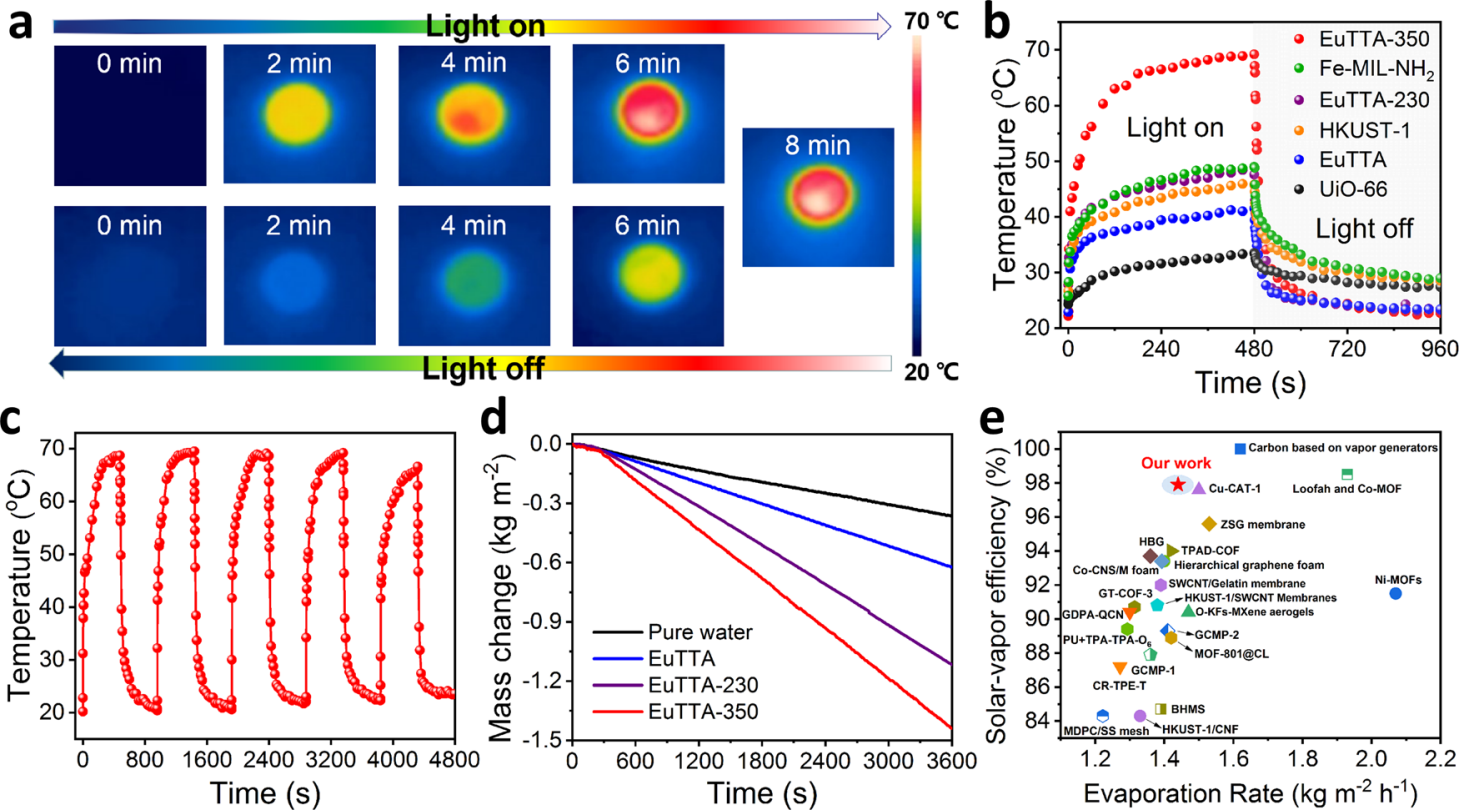
Characterization of photothermal conversion and water evaporation properties. **a** IR thermal images of EuTTA-350 powder (50 mg) under a Xenon lamp (1 kW m^−2^) and then with the Xenon lamp turned off. **b** Photothermal conversion behavior of EuTTA, EuTTA-230, EuTTA-350, Fe-MIL-NH_2_, HKUST−1, and UiO-66 powder samples under 1-sun irradiation within 480 s. **c** Anti-photobleaching property of EuTTA-350 powder during five cycles of heating–cooling. **d** Water evaporation under simulated sunlight with an intensity of 1 kW m^−2^ (1 sun). **e** Comparison of the performances among solar water evaporators based on various materials reported.

The radical-rich EuTTA-350 solid strongly absorbs across the broad visible/near-infrared regions (see Fig. 4b for the diffuse reflectance spectra), suggesting photothermal conversion uses. Also, the thermal conductivity of EuTTA-350 was found to be as low as 0.2 W K^−1^ m^−1^ (like that of rubber or mineral oil) at room temperature (Supplementary Fig. 41), indicating good thermal insulating properties suited for photothermal conversion applications. As is monitored by an infrared camera, the temperature of EuTTA-350 powder rises rapidly under a Xenon lamp (1 kW m^−2^, 420–2500 nm; to simulate 1-sun illumination): as shown in Fig. 5a, b, within 480 s, it increases by 47 °C to reach 69.2 °C, which compares favorably with other MOF materials^57,65–67^, second only to the reported values of Zr-Fc solid (Supplementary Fig. 43, the values of Zr-Fc-MOF measured in our hands are, however, lower than the reported numbers, e.g., rising by only 34.4 °C; we are currently investigating the reason for this disagreement). Notice that, under the same condition, our measured results for the known MOF solids of Fe-MIL-NH_2_, HKUST-1, and UiO-66 (Fig. 5b) are consistent with the relative literature values shown in Supplementary Fig. 43. By comparison, EuTTA and EuTTA-230 reach only 41.4 and 47.6 °C, respectively, under the same conditions. In addition, the photothermal performance of EuTTA-350 is stable: in all five illumination cycles tested, the temperature consistently rises to 65 °C within 480 s (Fig. 5c), and PXRD indicates the sample remains crystalline afterward (Supplementary Fig. 42).

So we use EuTTA-350 to build a solar-driven water evaporation device (Supplementary Fig. 48). As EuTTA-350 is light-weight and hydrophobic (with a high contact angle of 85.4°; Supplementary Fig. 49), its powder disperses to form a floating thin film, facilitate heat transfer to the water body for efficient interfacial evaporation. Under 1-sun (1 kW m^−2^) exposure in the air (using 50 mg of EuTTA-350), the evaporation rate (see the plot in Fig. 5d, see also plots in Supplementary Figs. 46 and 47 for other amounts of EuTTA-350) can reach 1.44 kg m^−2^ h^−1^, four times that of pure water (0.36 kg m^−2^ h^−1^), and also better than EuTTA (0.62 kg m^−2^ h^−1^) and EuTTA-230 (1.12 kg m^−2^ h^−1^), with the temperature equilibrating at 47.4 °C after 1 h (Supplementary Fig. 44). The infrared thermal image showed that the energy conversion takes place at the MOF layer (Supplementary Fig. 44). The solar-driven water evaporation efficiency is calculated to be 97.9%^68^, among the highest of all photothermal materials (Fig. 5e and Supplementary Table 3)^66,67,69–77^. Notice sample and device configurations are important factors, for example, entries 1, 2, and 4 in Supplementary Table 3 and many others depend on composite systems featuring nano-bristles or hierarchical particle sizes/surface areas to boost photothermal performances for water evaporation, whereas ours is simply a thin layer of powder floating on the water surface. This simple setup demonstrates the inherent strength of our system for photothermal applications; in other words, even higher performance metrics can likely be achieved in practical devices integrating the compound EuTTA-350.

In summary, the thermally induced ring contraction of the dithiin units to form thiophene and dithiole functions in the MOF solid of EuTTA is supported by both X-ray and NMR data (e.g., with the dithiole products in EuTTA-350 being evidenced by its tetrasulfonic derivative; Supplementary Fig. 33). The bonding/connection between the dithiole/thiophene units across the linker molecules, however, proves harder to characterize, because of the severe disorder in the crystal structures, and because of the likely reaction among the radical species once dissolved into solutions. Nevertheless, the persistence of the resulting radical species is notable, which, on a practical note, also imparts strongly visible/near IR absorption and enables efficient photothermal conversion for solar steam generation applications. On a broader front, advances can be envisioned from dithiin-equipped linker molecules (as exemplified here by TTA), as these offer a unique blend of stability (in air and water as protected S functions) and reactivity (from the ethene and the flanking S units) for material design. Building on this lead, one can integrate the dithiin function into other framework systems, in order to better synergize the interplay of porosity, open-shell/radical features, and crosslinks, and to open vistas for electronic and magnetic properties in the solid state.

## Methods

### Crystallization and activation of EuTTA

H_2_TTA (6.0 mg, 0.0173 mmol) and EuCl_3_·6H_2_O (8.4 mg, 0.023 mol) was loaded into a glass tube. Then a water/acetonitrile (4:1, 1 mL) mixed solution was added and the tube was sealed with an oxyhydrogen flame. The glass tube was placed in an oven at 150 °C for 48 h, during which yellow crystals slowly formed. After the sealed tube was heated for 48 h, it was cooled to room temperature for over 4 h. The yellow crystals were collected and washed with acetonitrile several times and air-dried to obtain yellow EuTTA crystals. For elemental analysis, the crystals were activated via Soxhlet extraction with methanol for 2 days, and then placed in a vacuum at 80 °C for 10 h. [C (31.65 %), H (1.24 %), S (24.62 %), N (0.29 %)]; a fitting formula can be determined to be Eu_2_(C_12_H_4_O_4_S_4_)_3_(H_2_O)_2_, which gives a calculated profile of [C (31.77 %), H (1.18 %), and S (28.26%)]. The synthesis of the new organic linker H_2_TTA can be found in Supplementary Fig. 1 and Supplementary Methods.

### Preparation of EuTTA-230 crystals

About 100 mg EuTTA as-made crystals were washed with acetonitrile (3 × 2 mL) and soaked in acetonitrile (3 × 3 mL, replaced by fresh acetonitrile after 12 h each time). The resulting crystals were filtered and then evacuated at 70 °C for 8 h. Then the crystal sample was heated in a tube furnace under a nitrogen atmosphere at 230 °C for 10 h. Heating rate: 5 °C/min.

### Preparation of EuTTA-350 crystals

The previous steps are the same as above. Then the crystal sample was heated in a tube furnace in a nitrogen atmosphere at 350 °C for 2 h. Heating rate: 5 °C/min.

### Photothermal conversion measurement

EuTTA-350 powder (70 mg) was spread on a quartz slide to form a thin circular film (thickness *ca*. 2 mm) with the largest possible surface area (diameter of 1.6 cm), which was set up at a distance of 30 cm from the Xenon lamp (AM 1.5 G, PLS-SXE300+), corresponding to an irradiance of 1 kW m^−2^ (1-Sun). An infrared camera was used to take infrared photographs of the EuTTA-350 MOF powder, e.g., when the illumination was turned on and off.

### Solar-driven vapor generation experiments

The EuTTA-350 powder was floated on water in a quartz beaker (see Supplementary Fig. 44). Sunlight was simulated by a Xenon lamp (PLS-SXE300+) with an optical filter (AM 1.5 G) and used to irradiate the sample under specific power density. The mass change of the water was recorded by an electronic balance (accuracy of 0.00001 g). An IR camera was used to measure the temperature. All experiments were conducted at an ambient temperature of 22 °C and a humidity of 65%.

## Supplementary information

Supplementary Information

## Data Availability

The data that support the findings of this study are available within the paper, and its supplementary information files or are available from the corresponding authors upon request. Source data are provided with this paper. The X-ray crystallographic coordinates for structures reported in this study have been deposited at the Cambridge Crystallographic Data Center (CCDC), under the deposition numbers: 2132960 (EuTTA) and 2132961 (EuTTA-230). These data can be obtained free of charge from The Cambridge Crystallographic Data Center via www.ccdc.cam.ac.uk/data_request/cif. Source data are provided with this paper.

## Source data

Source Data
