# Supplementary material for: Organic radicals stabilization above 300 °C in Eu-based coordination polymers for solar steam generation
Source: Nat Commun. 2022 Oct 17;13:6116. doi: 10.1038/s41467-022-33948-9 (PMC9576730; doi:10.1038/s41467-022-33948-9)
Supplement: Supplementary file 1 — Supplementary Information [file 41467_2022_33948_MOESM1_ESM.pdf]

Supplementary Information

Organic radicals stabilization above 300 °C in Eu-based coordination polymers for solar steam generation

Xinhe Ye[1], Lai-Hon Chung[1], Kedi Li[2], Saili Zheng[1], Yan-Lung Wong[2], Zihao Feng[1], Yonghe He[1], Dandan Chu[2], Zhengtao Xu[*,3], Lin Yu[1] & Jun He[*,1]

[1] School of Chemical Engineering and Light Industry, Guangdong University of Technology, Guangzhou 510006, China.

[2] Department of Chemistry, City University of Hong Kong, 83 Tat Chee Avenue, Kowloon, Hong Kong, China.

[3] Institute of Materials Research and Engineering (IMRE), Agency of Science, Technology and Research (A*STAR), 2 Fusionopolis Way, Singapore 138634, Republic of Singapore.

* Email: junhe@gdut.edu.cn; zhengtao@imre.a-star.edu.sg

Table of contents

Supplementary Methods. S4

Supplementary Figure 1. The synthetic scheme for H_2TTA. S5

Supplementary Figure 2. The synthetic scheme for *anti*-BDT S7

Supplementary Discussion. S9

Supplementary Figure 3-19. ^1H and ^{13}C NMR, IR and MS spectra of the ligand H_2TTA, *anti*-BDT and intermediates. S9-S17

Supplementary Figure 20. The IR spectra of H_2TTA and an as-made sample of EuTTA. S17

Supplementary Figure 21. PXRD patterns of EuTTA immersed in aqueous solutions with different pH values from 1 to 13 for 2 hours. S17

Supplementary Figure 22. Two thermogravimetric analysis (TGA) plots of EuTTA. S18

Supplementary Figure 23. PXRD patterns and IR spectra of EuTTA heating at different temperatures. S18

Supplementary Figure 24. Photographs of crystals of EuTTA, EuTTA-230 and EuTTA-350. S18

Supplementary Figure 25-26. Adsorption and desorption isotherms of EuTTA, EuTTA-300 and EuTTA-350 for N_2 at 77 K, CO_2 at 195 K and 273 K. S19

Supplementary Figure 27. Solution ^1H NMR spectra of as-made EuTTA, EuTTA-230 and EuTTA-350 crystals dissolved in HF/DMSO-d_6. S20

Supplementary Figure 28. Solid-state electron paramagnetic resonance (EPR) spectra of EuTTA, EuTTA-230 and EuTTA-350 powders. S21

Supplementary Figure 29. Solid-state electron paramagnetic resonance (EPR) spectra of the organic components extracted from EuTTA-350. S21

Supplementary Figure 30-31. The XPS spectra of EuTTA and EuTTA-350. S22-23

Supplementary Figure 32. Normalized diffuse reflectance spectra and Kubelka–Munk plots of EuTTA, EuTTA-230 and EuTTA-350 powders. S24

Supplementary Figure 33. The ^1H and ^{13}C NMR spectra of the H_2O_2-oxidized soluble components of the EuTTA-350 crystal. S25

Supplementary Figure 34. Molecular weight distribution by GPC of EuTTA-350 after dissolution in DMSO/HF. S26

Supplementary Figure 35. Thermogravimetry, gas chromatography, mass spectrometry (TG-GC-MS) S26

Supplementary Figure 36-39. PXRD, IR, and elemental mapping by EDS of the insoluble black solid after dissolving the bulk of EuTTA-350 by HF/DMSO-d_6. S27-S28

Supplementary Figure 40. A ChemDraw scheme for illustrating the crosslinking S29

across the linker species in the 350 °C-treated sample EuTTA-350.
Supplementary Figure 41. Variation of the thermal conductivity of EuTTA-350 with respect to the temperature (from 50-350 K). S30
Computational details. S30-S32
Supplementary Table 1. Cartesian coordinates, Mulliken charge and spin density of bis(dithiole). S31
Supplementary Table 2. Spin natural orbital (SNO) contribution of two spins on bis(dithiole). S31-S32
Supplementary Figure 42. PXRD patterns of EuTTA-350 after 5 photothermal conversion cycles. S33
Supplementary Figure 43. A plot of the maximum temperature rises and the corresponding time (in seconds) entailed for various photothermal materials. S33
Supplementary Figure 44. The optical photographs and the corresponding IR photographs of an EuTTA-350 powder floating on the water surface. S34
Supplementary Figure 45. A bar chart for water evaporation rates under xenon lamp irradiation and without xenon lamp irradiation. S34
Calculation of the efficiency for solar to vapor generation. S35-S36
Supplementary Figure 46. A plot of mass loss of water against time under different mass of EuTTA-350 powder. S36
Supplementary Figure 47. Water evaporation rates under different mass of EuTTA-350 powder. S37
Supplementary Figure 48. Photographs for the device for recording the water mass change under solar irradiation. S37
Supplementary Figure 49. Photographs for the water contact angle measurement of a pressed pellet of a sample of EuTTA and EuTTA-350. S37
Supplementary Table 3. Performance metrics of reported materials for solar-driving water evaporation. S38-S39
Supplementary Table 4. Crystal Data and Structure Refinement Parameters for EuTTA and EuTTA-230. S40
Supplementary References. S41-S42

Supplementary Methods

General procedure

Starting materials, reagents, and solvents were purchased from commercial sources (J&K, Zhengzhou Alfa and Acros) and used without further purification. Elemental analysis was obtained with a Vario Micro CUBE CHN elemental analyzer. Powder X-ray diffraction (PXRD) patterns were collected on a Rigaku Smart lab diffractometer with Cu Kα radiation (λ = 1.5418 Å) at room temperature. The X-ray tube operated at a voltage of 40 kV and a current of 15 mA. FT-IR spectra in the range 400-4000 cm^{-1} were recorded on a Nicolet Avatar 360 FT-IR spectrophotometer. Solution ^{1}H NMR and ^{13}C NMR spectra were recorded on a 400 MHz Bruker superconducting magnet high-field NMR spectrometer at 298 K, with tetramethylsilane (TMS) as the internal standard. Chemical shifts (δ) are expressed in ppm relative to the residual solvent (*e.g.,* chloroform ^{1}H: 7.26 ppm, ^{13}C: 77.0 ppm) reference. Coupling constants are expressed in hertz. Thermogravimetric analyses (TGA) were carried out in a PerkinElmer thermal analysis equipment (STA 6000). X-ray photoelectron spectroscopy (XPS) measurements were conducted on a Thermo Fisher XPS system (Escalab 250Xi) with a monochromatic Al Kα X-ray source. Single crystal X-ray diffraction data of EuTTA as-made sample was collected on a Bruker APEX-II CCD single diffractometer (Cu Kα radiation, λ = 1.54178 Å) at T = 150 K. Single crystal X-ray diffraction data of EuTTA-230 sample was collected on a Bruker APEX-II CCD single diffractometer (Cu Kα radiation, λ = 1.54184 Å) at T = 230 K. Diffuse reflection spectrum was collected in the UV-Visible Near Infra-Red Spectrophotometer with Integrating Sphere (PE Lamda 750). All of the magnetic and thermal conductivity data were obtained using a physical property measurement system (PPMS, DynaCool, Quantum Design Inc). The electron paramagnetic resonance (EPR) spectrum was obtained at 300 K using a Bruker ER-420 spectrometer. The central magnetic field was 2890 G, and the modulation frequency was 100 kHz. The morphology of the samples was investigated using scanning electron microscopy (SEM, SIGMA 500, ZEISS, Germany). Thermogravimetry, gas chromatography, mass spectrometry (TG-GC-MS) were carried out in Mettler Toledo TGA2 equipment, IST16 sample storage unit and Agilent 7890B-5977B Gas Chromatography Mass Spectrometry. The molecular mass distributions (Mw/Mn) of the samples were measured by gel permeation chromatography (Agilent, PL-GPC 50) at 40°C using DMSO as a solvent.

Ligand synthesis

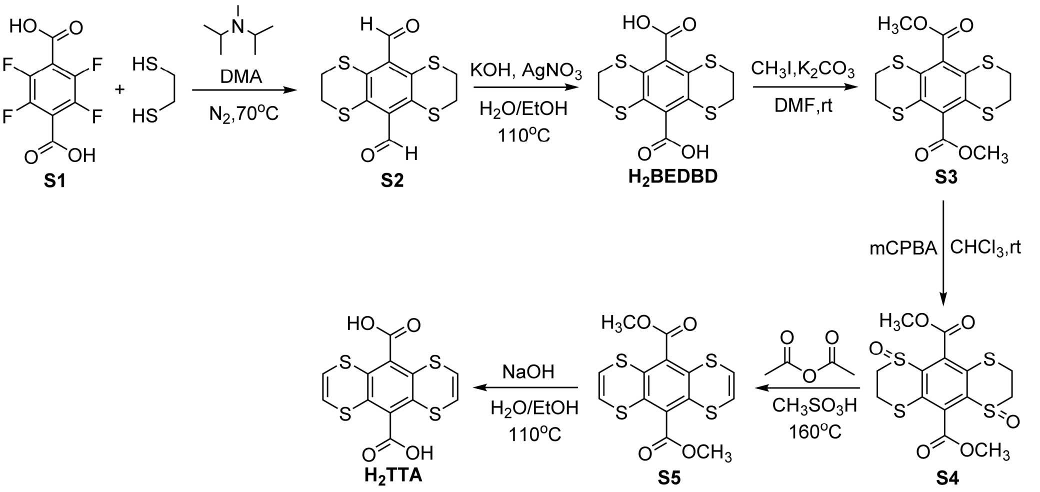

Supplementary Figure 1. The synthetic scheme for H_2TTA.

Synthesis of S2. The starting material S1 (2.0 g, 9.71 mmol) and diisopropylethylamine (6.5 g, 50.29 mmol) were stirred in *N,N*-dimethylacetamide (DMA, 20.0 mL, purged with N_2 for 5 minutes beforehand) in a 100-mL two-neck, round-bottom flask. Ethylene dithiol (2.0 mg, 21.23 mmol) was then added under N_2 protection. The mixture was stirred at 70 °C for 12 hours during which the reaction mixture turned from a light-yellow clear solution to a cloudy red mixture. After cooling to room temperature, the resulting red mixture was poured into distilled water (200 mL) and a red precipitate thus formed was collected by suction filtration, washed with distilled water (3 × 200 mL) and suction-dried to afford an orange crude solid (3.15 g). Purification of the crude product by flash column chromatography (eluent: dichloromethane (DCM)/*n*-hexane, v/v = 2:1) afforded S2 as a crystalline red solid (2.15 g, 70.4% based on S1) Solution ^{1}H NMR (400 MHz, DMSO-d_6): δ = 10.26 (s, 2H, C*H*O), 3.27 (s, 8H). Solution ^{13}C NMR (100 MHz, DMSO-d_6): δ = 190.18, 137.03, 136.06, 31.94.

Synthesis of H_2BEDBD. Silver nitrate (543 mg, 3.20 mmol) and S2 (200 mg, 0.64 mmol) were loaded into a 100-mL round-bottom flask. Distilled water (30.0 mL) was then added and the mixture was stirred at 110 °C for 30 minutes. Afterward, aqueous potassium hydroxide (180 mg, 3.21 mmol) was added to the stirring solution and the flask was connected with a water condenser. The mixture was heated in an oil bath at 100 °C for 5 hours. Afterward, the reaction mixture was cooled to room temperature. The resulting black mixture was acidified with 2%

HNO_3 (aq) to attain a pH of 2 as indicated by pH paper. The black solid residues were collected by suction filtration and washed with distilled water (3 × 100 mL). The solid mixture thus obtained was stirred in 0.5% NaOH (aq) (500 mL) at room temperature in order to dissolve the desired linker product. The resulting deep brown mixture was filtered and the brown filtrate was acidified with 2% HNO_3 (aq). The white-off precipitate was collected by suction filtration, washed with distilled water (3 × 100 mL), and suction-dried to afford H_2BEDBD as a grey solid (176 mg, 80% based on S2). Solution ^{1}H NMR (300 MHz, DMSO-d_6): δ = 3.21(s, 8H). Solution ^{13}C NMR (100 MHz, DMSO-d_6): δ = 167.23, 136.71, 129.17, 30.52.

Synthesis of S3. H_2BEDBD (176 mg, 0.51 mmol) and potassium carbonate (123 mg, 0.89 mmol) were loaded into a 7.5-mL clear glass vial. *N,N*-Dimethylforamide (DMF, 5.0 mL) was added and the stirring was started. Methyl iodide (690 mg, 4.86 mmol) was added and the reaction mixture was kept stirring at room temperature for 24 hours. Afterward, the resulting mixture was poured into distilled water (20 mL) and the white precipitate thus formed was collected by suction filtration, washed with distilled water (3 × 10 mL) and suction-dried to afford S3 as a white solid (191 mg, 100% based on H_2BEDBD). Solution ^{1}H NMR (400 MHz, DMSO-d_6): δ = 3.96 (s, 6H, $COOCH_3$), 3.19 (s, 8H). Solution ^{13}C NMR (100 MHz, DMSO-d_6): δ = 166.32, 135.42, 130.10, 53.20, 30.42.

Synthesis of S4. 3-Chloroperbenzoic acid (*m*-CPBA, 114 mg, 0.66 mmol) was dissolved in chloroform (3.0 mL) and added dropwise over 5 minutes into a stirring chloroform solution (2.0 mL) of S3 (113 mg, 0.30 mmol) at room temperature. After 15 minutes, the chloroform solvent was removed by rotary evaporation and the solid residue was purified by flash column chromatography (eluent: DCM/ethyl acetate, v/v = 3:1). The purified product was a white solid S4**.** However, the NMR spectrum showed mixture of species probably stemming from several isomers with oxygen on different position but comparable polarity and could not be resolved by column chromatography. Most importantly, dehydration using S4 gave one product showing pure NMR signals corresponding to S5.

Synthesis of S5. Acetic anhydride (3.0 mL) was added to a glass vial containing S4 (158.4 mg, 0.39 mmol) and stirring was started. A drop of methanesulfonic acid (CH_3SO_3H) was added and the glass vial was screw-capped and the mixture was stirred at 160 °C for 15 minutes. After cooling to room temperature, the mixture was poured into DCM (10 mL) and shaken with aqueous $NaHCO_3$ solution (3 × 10 mL). The DCM fraction was collected, followed by solvent removal through rotary evaporation and the resulting solid residue was purified by flash column chromatography (eluent: DCM/*n*-hexane, v/v = 1:2). The purified product was a yellow solid S5 (73 mg, 50% based on S4). Solution ^{1}H NMR (400 MHz, $CDCl_3$): δ = 4.02 (s, 6H, $COOCH_3$), 6.58 (s, 4H). Solution ^{13}C NMR (100 MHz, $CDCl_3$): δ = 165.58, 133.58, 133.16, 125.48, 53.03.

Synthesis of H_2TTA (1, 4, 5, 8-tetrathiaanthracene-9,10-dicarboxylic acid). A binary solvent mixture of NaOH (17.1 mmol of NaOH in EtOH/H_2O, v/v = 1:1, 30 mL) was added to a 100-mL round-bottom flask containing S5 (158 mg, 0.43 mmol) and stirring was started. The flask was then connected to a water condenser and heated in an oil bath at 110 °C for 2 days. After cooling to room temperature, the resulting mixture was poured into distilled water (50 mL) and acidified with 10% aqueous HCl to attain a pH of 2. A white precipitate was collected by suction filtration, washed by distilled water (3 × 100 mL) and dried in an oven at 50 °C for 2 hours. Yield 125 mg (85% based on S5). Solution ^{1}H NMR (400 MHz, DMSO-d_6): δ = 6.88 (s, 4H). Solution ^{13}C NMR (100 MHz, DMSO-d_6): δ = 166.58, 134.84, 131.61, 125.74.

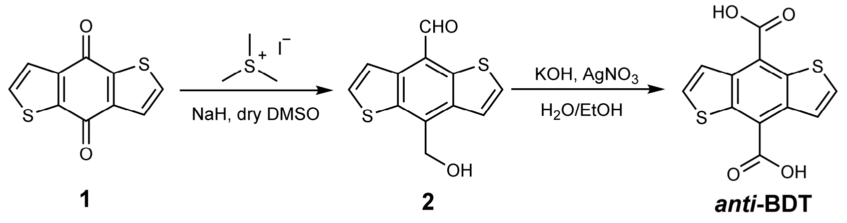

Supplementary Figure 2. The synthetic scheme for *anti*-BDT.

Synthesis of 8-Hydroxymethylbenzo[1,2-b:4,5-b']dithiophene-4-carboxaldehyde (2). Dried trimethylsulfonium iodide (1.06 g, 5.2 mmol; note: drying this agent over vacuum drying oven is important for this reaction) was added to sodium hydride 223 mg of 60% in oil washed with 3 × 100 ml of cyclohexane) in 4 ml of dry DMSO and stirred for 1 hour in subdued light.

To this was added dropwise over 20 minutes a solution of benzo[1,2-b:4,5-b']dithiophene-4,8-dione (500 mg, 2.27 mmol) in 4 mL of dry DMSO. Stirring at room temperature was continued for 12 hours after which the mixture was poured into ice/water (w/w = 2:1) mixture. The solid was collected, washed with water and dried. Purification of the crude product by flash column chromatography (eluent: ethyl acetate/*n*-hexane, v/v = 1:6) afforded 2 as a crystalline orange solid (168 g, 29.8% based on 1). Solution ^{1}H NMR (400 MHz, DMSO-d_6): δ = 10.97 (s, 1H, C*H*O), 8.36 (d, 1H), 8.16 (d, 1H), 7.95 (d, 1H), 7.80 (d, 1H), 5.92 (t, 3H, OH), 5.26 (d, 2H). Solution ^{13}C NMR (100 MHz, DMSO-d_6): δ = 190.33, 141.57, 137.92, 136.64, 136.47, 135.78, 133.76, 131.32, 122.11, 120.91, 119.55, 61.74. ESI-MS m/z (%): calcd., 249.0 (100%) for [$(M+H)^+$]; found, 249.0 $(M + H)^+$.

Synthesis of Benzo[1,2-b:4,5-b']dithiophene-4,8-dicarboxylic acid (*anti*-BDT). Silver nitrate (1.1 g, 6.5 mmol) and **2** (200 mg, 0.81 mmol) were loaded into a 25-mL round-bottom flask. Distilled water/ethanol (8 mL, v/v = 1:1) was then added and the mixture was stirred at 110 °C for 30 minutes. Afterward, an aqueous solution of potassium hydroxide (903 mg, 16 mmol) was added to the stirring solution and the flask was fitted with a water condenser. The mixture was heated in an oil bath at 110 °C for 12 hours. Afterward, the reaction mixture was cooled to room temperature. The resulting faint yellow mixture was filtered and the faint yellow filtrate was acidified with 68% HNO_3 to give orange precipitates which were collected by suction filtration, washed by distilled water (3 × 100 mL), and suction-dried to afford *anti*-BDT as orange solid (198 mg, 88.4% based on 2). Solution ^{1}H NMR (400 MHz, DMSO-d_6): δ = 8.27 (d, 2H), 8.05 (d, 2H). Solution ^{13}C NMR (100 MHz, DMSO-d_6): δ = 167.81, 140.98, 137.87, 133.61, 123.69, 122.43. ESI-MS m/z (%): calcd., 276.97 (100%) for $(M-H)^-$; found, 276.96 $(M–H)^-$.

Supplementary Discussion.

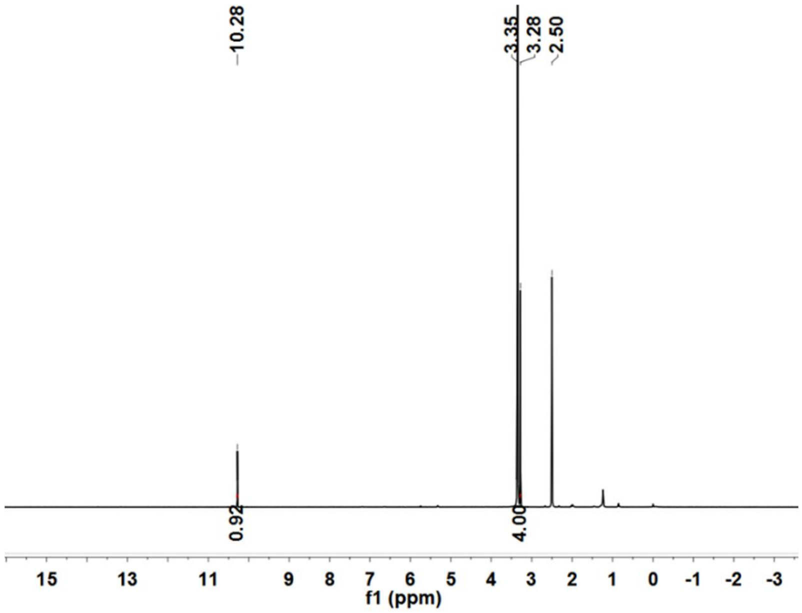

Supplementary Figure 3. The ^{1}H NMR spectrum of compound S2 in DMSO-d_6. The spectrum was collected on a 400 MHz Bruker superconducting magnet high-field NMR spectrometer at 298 K.

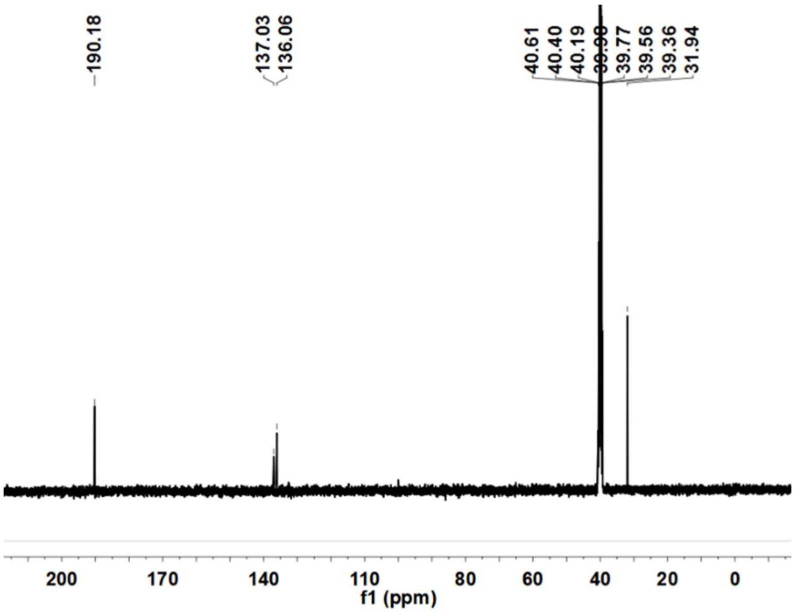

Supplementary Figure 4. The ^{13}C NMR spectrum of compound S2 in DMSO-d_6. The spectrum was collected on a 400 MHz Bruker superconducting magnet high-field NMR spectrometer at 298 K.

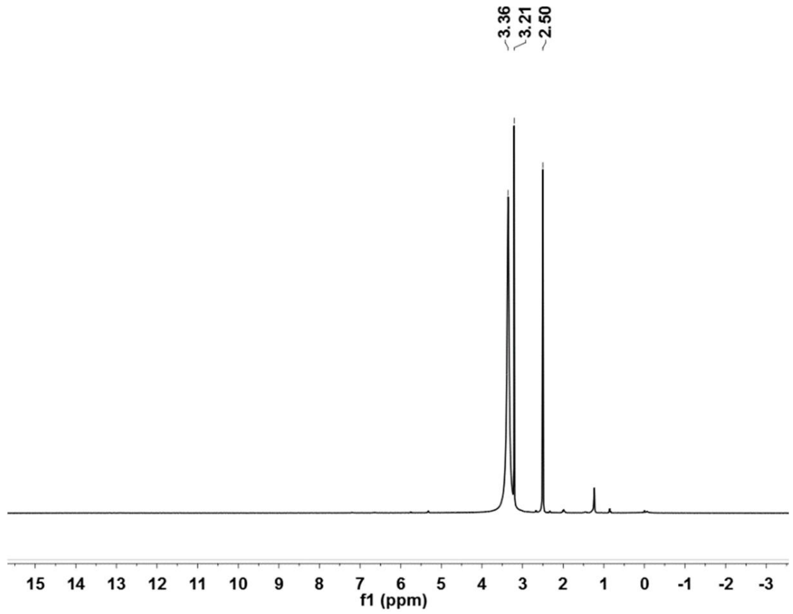

Supplementary Figure 5. The ^{1}H NMR spectrum of compound H_2BEDBD in DMSO-d_6. The spectrum was collected on a 400 MHz Bruker superconducting magnet high-field NMR spectrometer at 298 K.

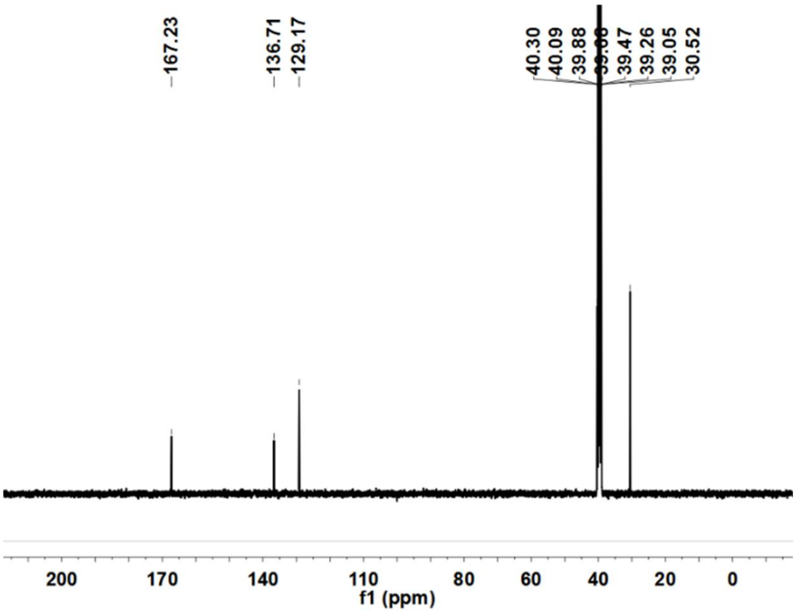

Supplementary Figure 6. The ^{13}C NMR spectrum of compound H_2BEDBD in DMSO-d_6. The spectrum was collected on a 400 MHz Bruker superconducting magnet high-field NMR spectrometer at 298 K.

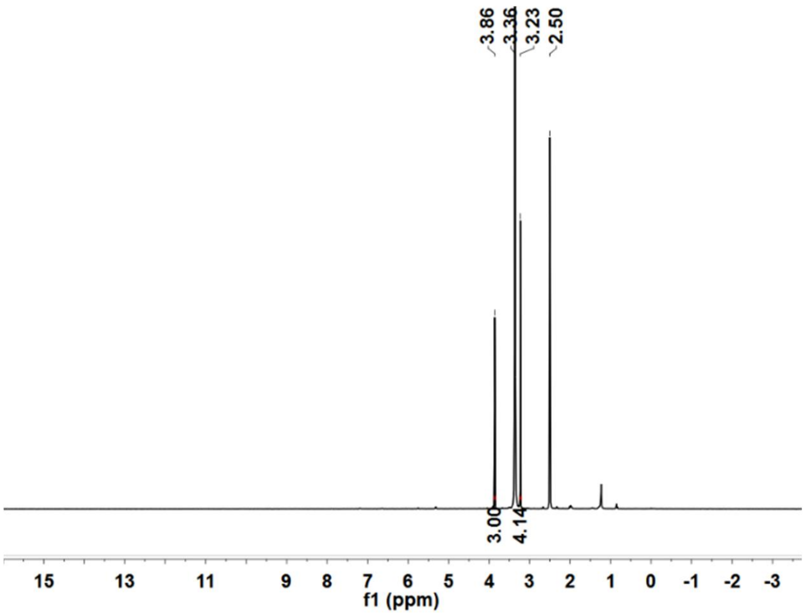

Supplementary Figure 7. The ^{1}H NMR spectrum of compound S3 in DMSO-d_6. The spectrum was collected on a 400 MHz Bruker superconducting magnet high-field NMR spectrometer at 298 K.

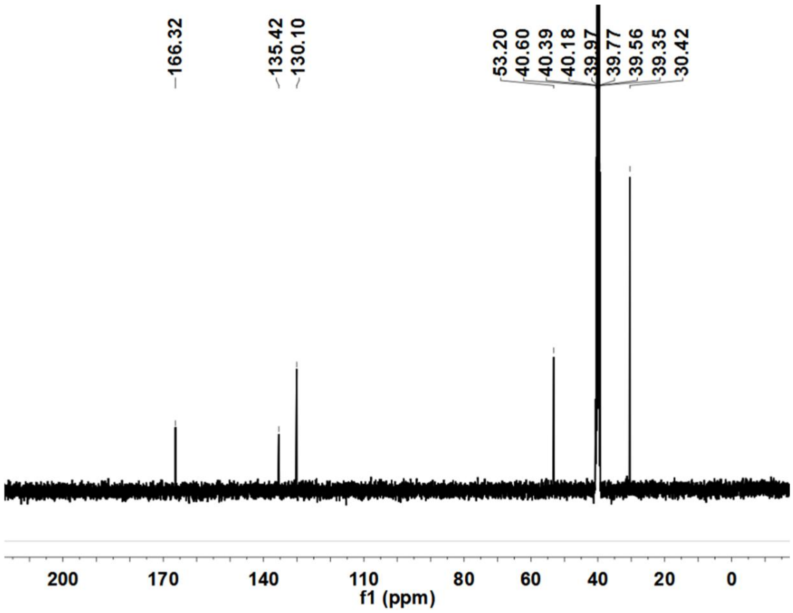

Supplementary Figure 8. The ^{13}C NMR spectrum of compound S3 in DMSO-d_6. The spectrum was collected on a 400 MHz Bruker superconducting magnet high-field NMR spectrometer at 298 K.

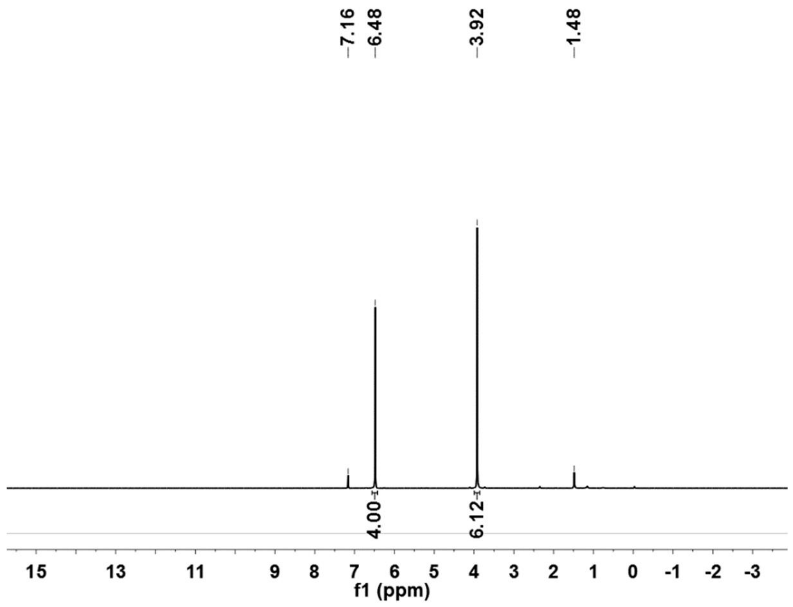

Supplementary Figure 9. The ^{1}H NMR spectrum of compound S5 in $CDCl_3$. The spectrum was collected on a 400 MHz Bruker superconducting magnet high-field NMR spectrometer at 298 K.

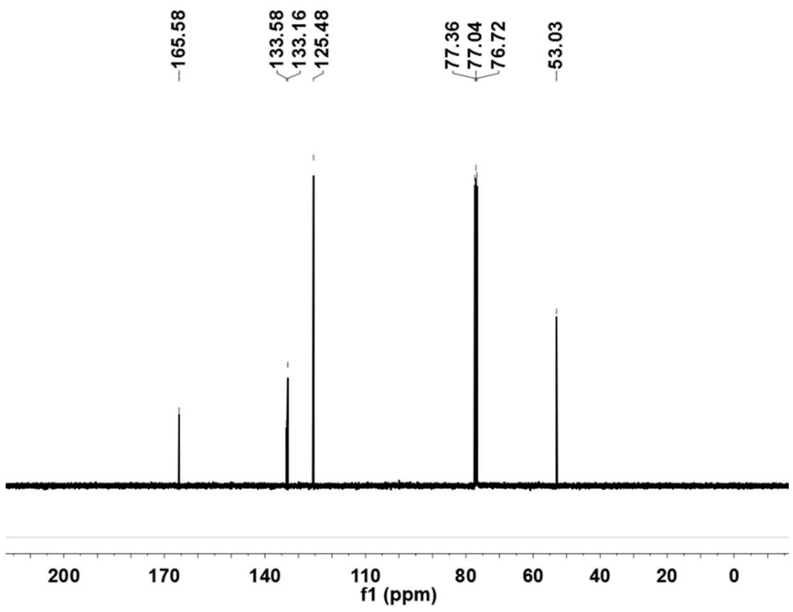

Supplementary Figure 10. The ^{13}C NMR spectrum of compound S5 in $CDCl_3$. The spectrum was collected on a 400 MHz Bruker superconducting magnet high-field NMR spectrometer at 298 K.

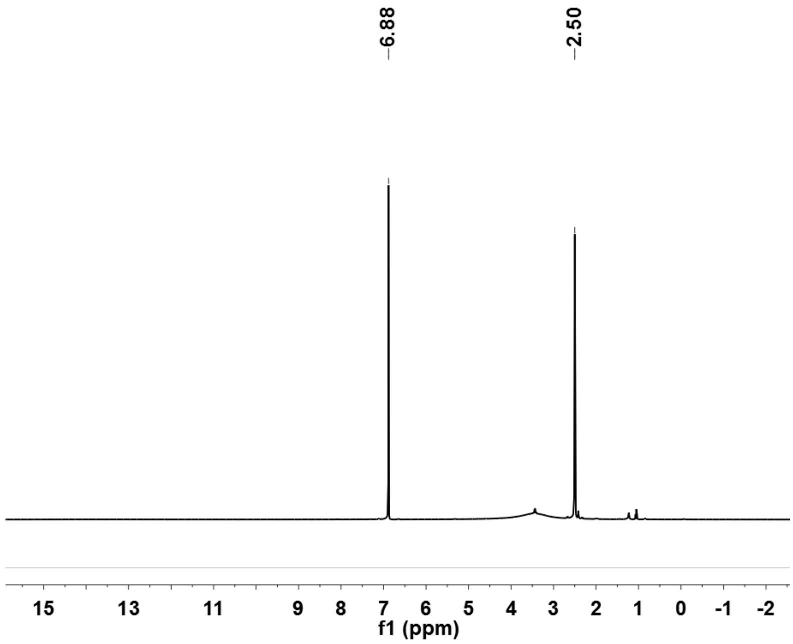

Supplementary Figure 11. The ¹H NMR spectrum of compound H_2TTA in DMSO-d_6. The spectrum was collected on a 400 MHz Bruker superconducting magnet high-field NMR spectrometer at 298 K.

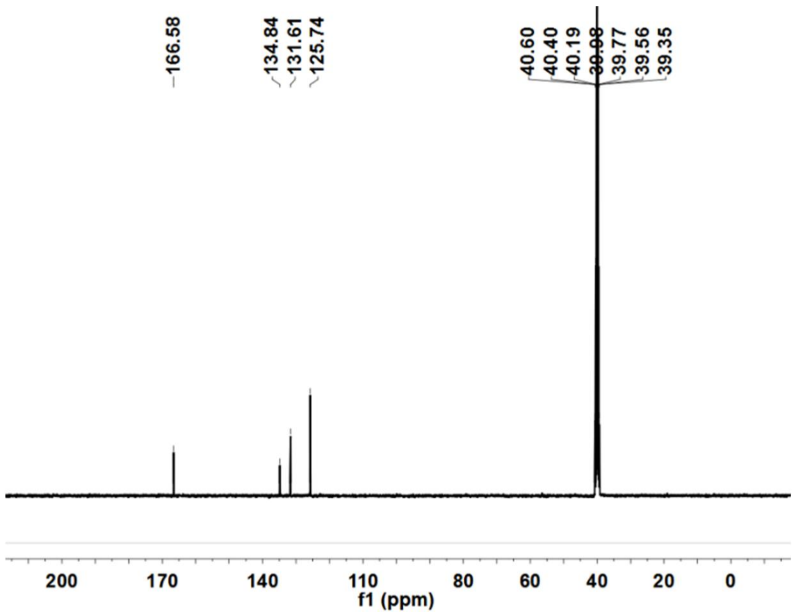

Supplementary Figure 12. The ¹³C NMR spectrum of compound H_2TTA in DMSO-d_6. The spectrum was collected on a 400 MHz Bruker superconducting magnet high-field NMR spectrometer at 298 K.

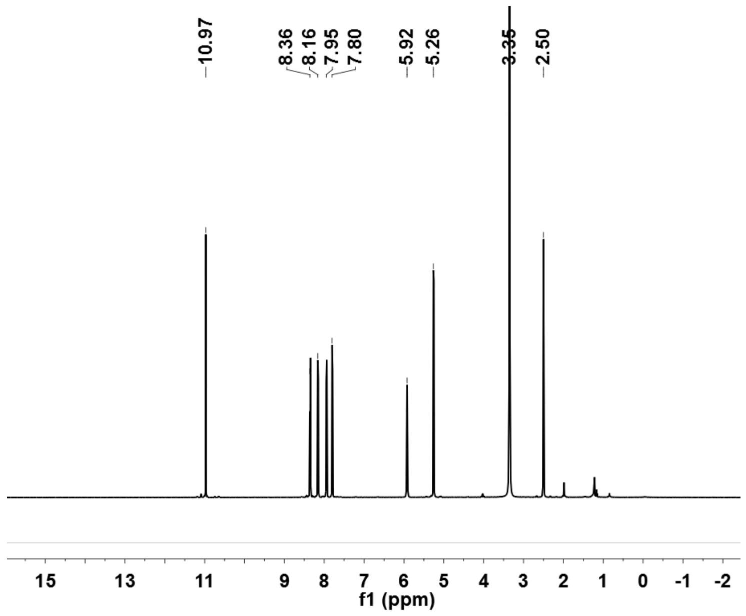

Supplementary Figure 13. The ^{1}H NMR spectrum of compound 8-Hydroxymethylbenzo[1,2-b:4,5-b']dithiophene-4-carboxaldehyde (**2**) in DMSO-d_6. The spectrum was collected on a 400 MHz Bruker superconducting magnet high-field NMR spectrometer at 298 K.

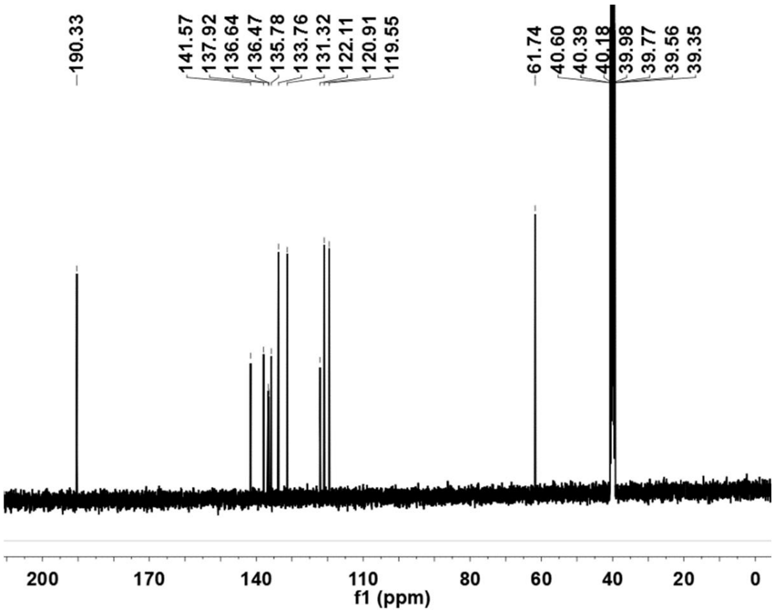

Supplementary Figure 14. The ^{13}C NMR spectrum of compound 8-Hydroxymethylbenzo[1,2-b:4,5-b']dithiophene-4-carboxaldehyde (**2**) in DMSO-d_6. The spectrum was collected on a 400 MHz Bruker superconducting magnet high-field NMR spectrometer at 298 K.

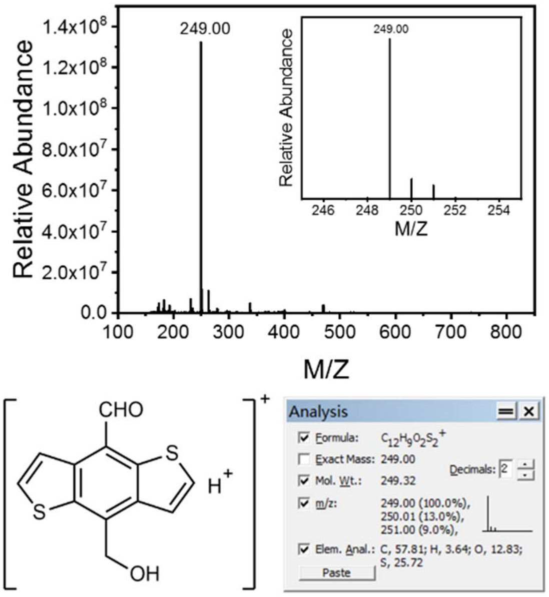

Supplementary Figure 15. A mass spectrum (ESI) of compound 8-Hydroxymethylbenzo[1,2-b:4,5-b']dithiophene-4-carboxaldehyde (2), m/z (%): calcd., 240.0 for the pseudo molecular ion $(M+H)^+$ (100%); found, 249.00 (100%) $(M+H)^+$.

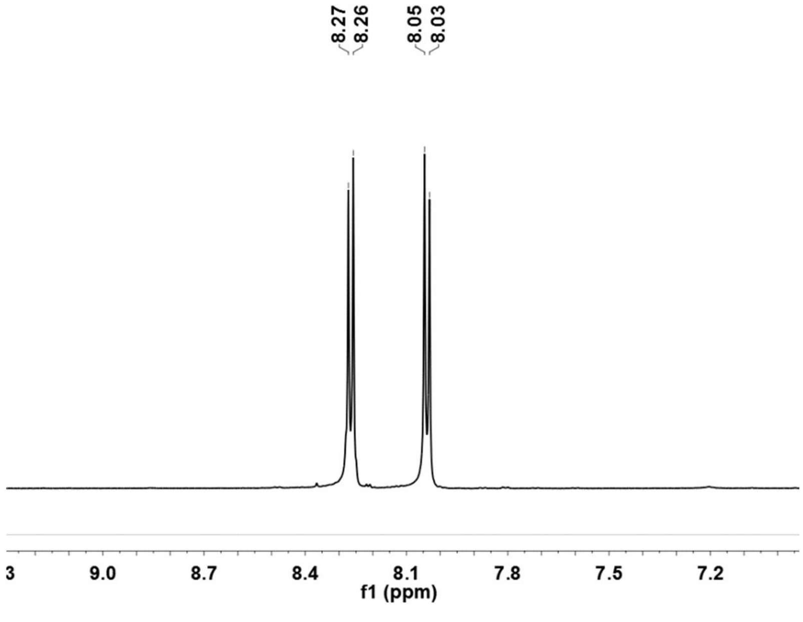

Supplementary Figure 16. The ^{1}H NMR spectrum of compound *anti*-BDT in DMSO-d_6. The spectrum was collected on a 400 MHz Bruker superconducting magnet high-field NMR spectrometer at 298 K.

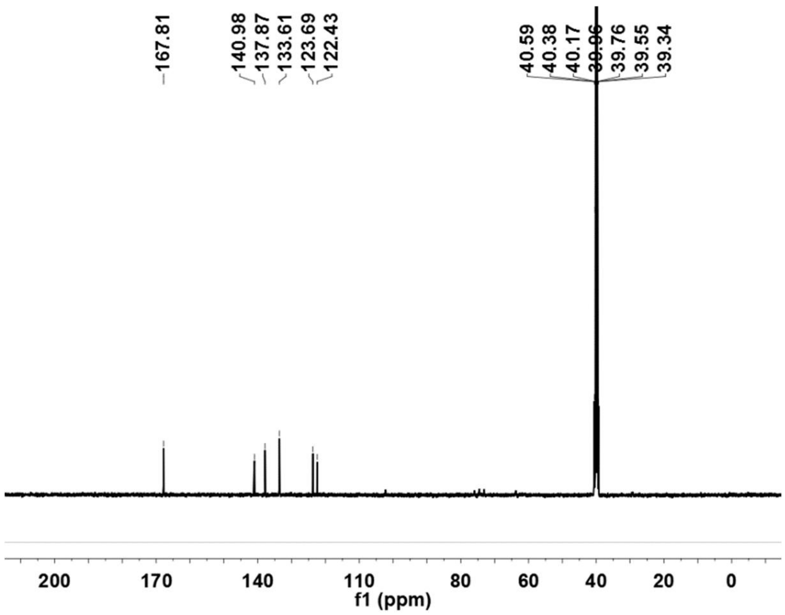

Supplementary Figure 17. The ^{13}C NMR spectrum of compound *anti*-BDT in DMSO-d_6. The spectrum was collected on a 400 MHz Bruker superconducting magnet high-field NMR spectrometer at 298 K.

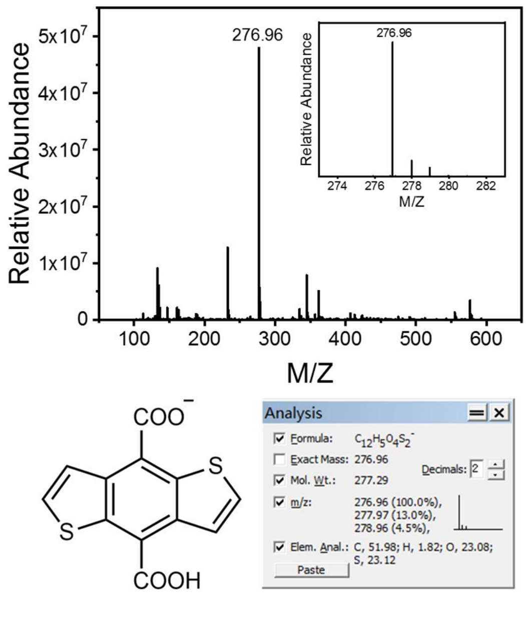

Supplementary Figure 18. A mass spectrum (ESI) of compound *anti*-BDT, m/z (%): calcd., 276.96 for the pseudo molecular ion (M-H)$^-$ (100%); found, 276.96 (100%) (M-H)$^-$.

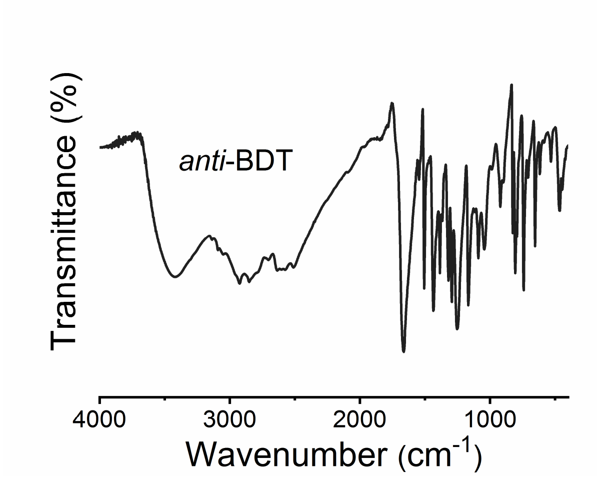

Supplementary Figure 19. The IR spectrum of *anti*-BDT.

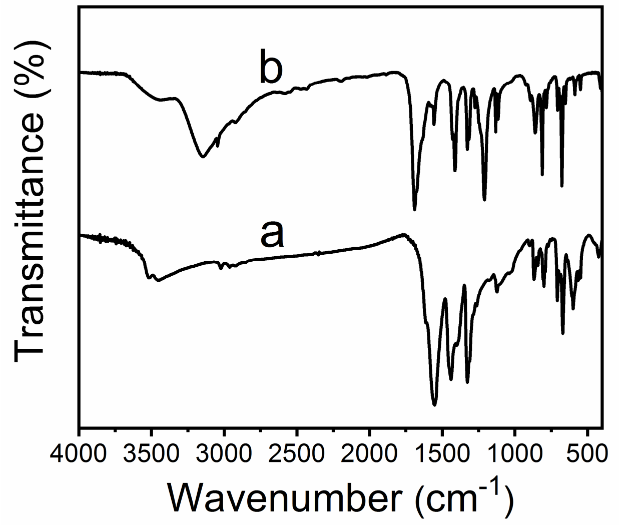

Supplementary Figure 20. The IR spectra of a) an as-made sample of EuTTA; b) H_2TTA.

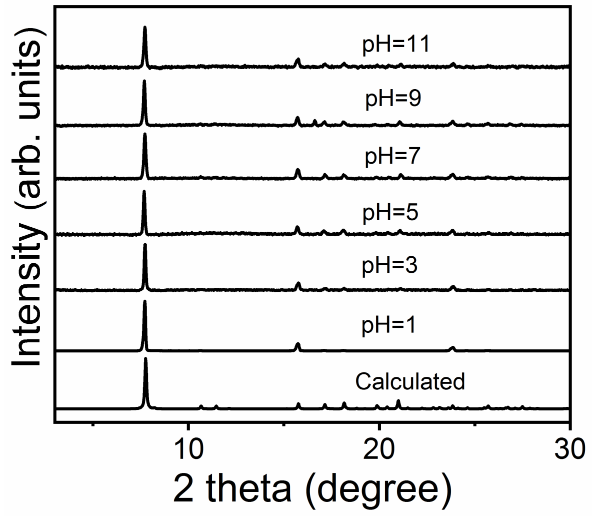

Supplementary Figure 21. PXRD patterns of EuTTA immersed in aqueous solutions with different pH values from 1 to 13 for 2 hours.

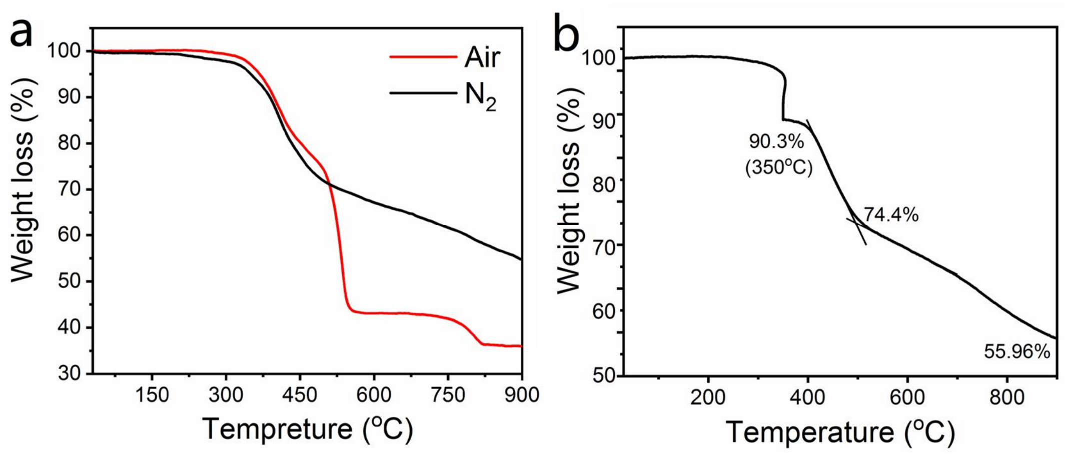

Supplementary Figure 22. Two thermogravimetric analysis (TGA) plots of EuTTA. Temperature programs: a) heating from 30 °C to 900 °C at 2 °C/minute; b) heating from 30 °C to 350 °C, then stay at 350 °C for 2 hours, then heat to 900 °C at 2 °C/minute.

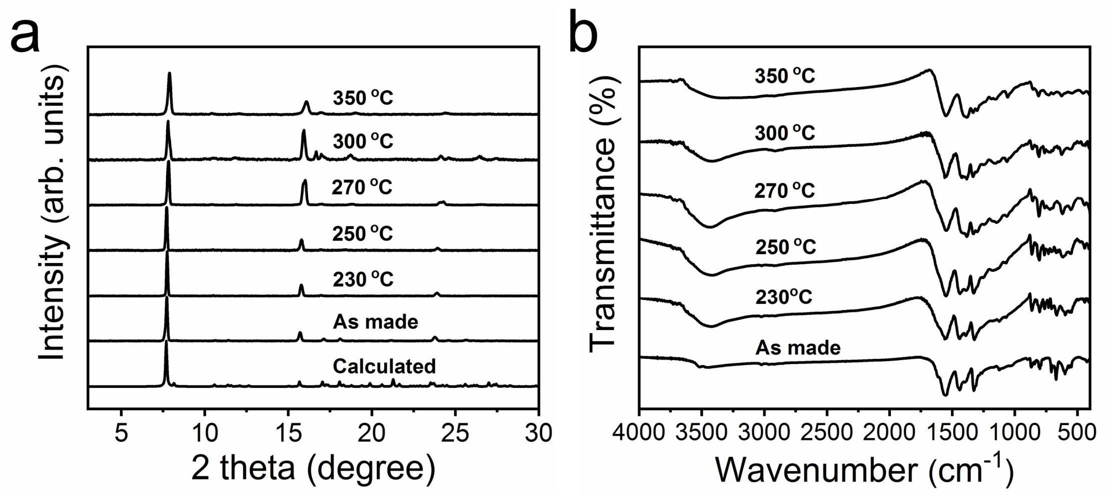

Supplementary Figure 23. (a) PXRD patterns and (b) IR spectra of EuTTA heating at different temperatures.

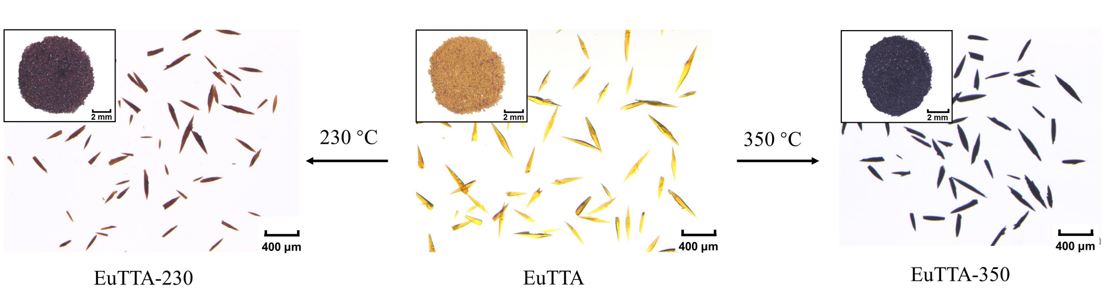

Supplementary Figure 24. Photographs of crystals of EuTTA, EuTTA-230 and EuTTA-350 under optical microscope (the scale bar is 400 μm), with insets showing the photographs of the corresponding bulk samples (the scale bar is 2 mm)

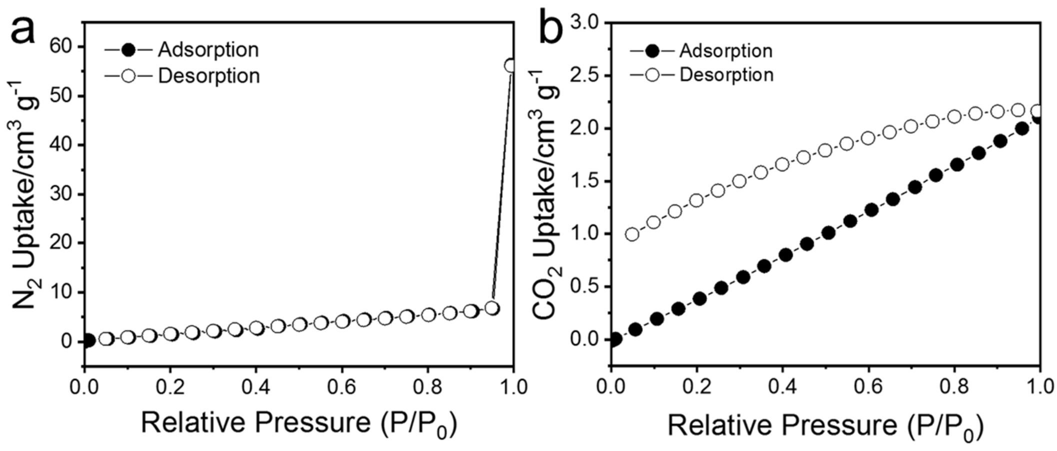

Supplementary Figure 25. Adsorption and desorption isotherms of EuTTA for (a) N_2 at 77 K, and (b) CO_2 at 195 K.

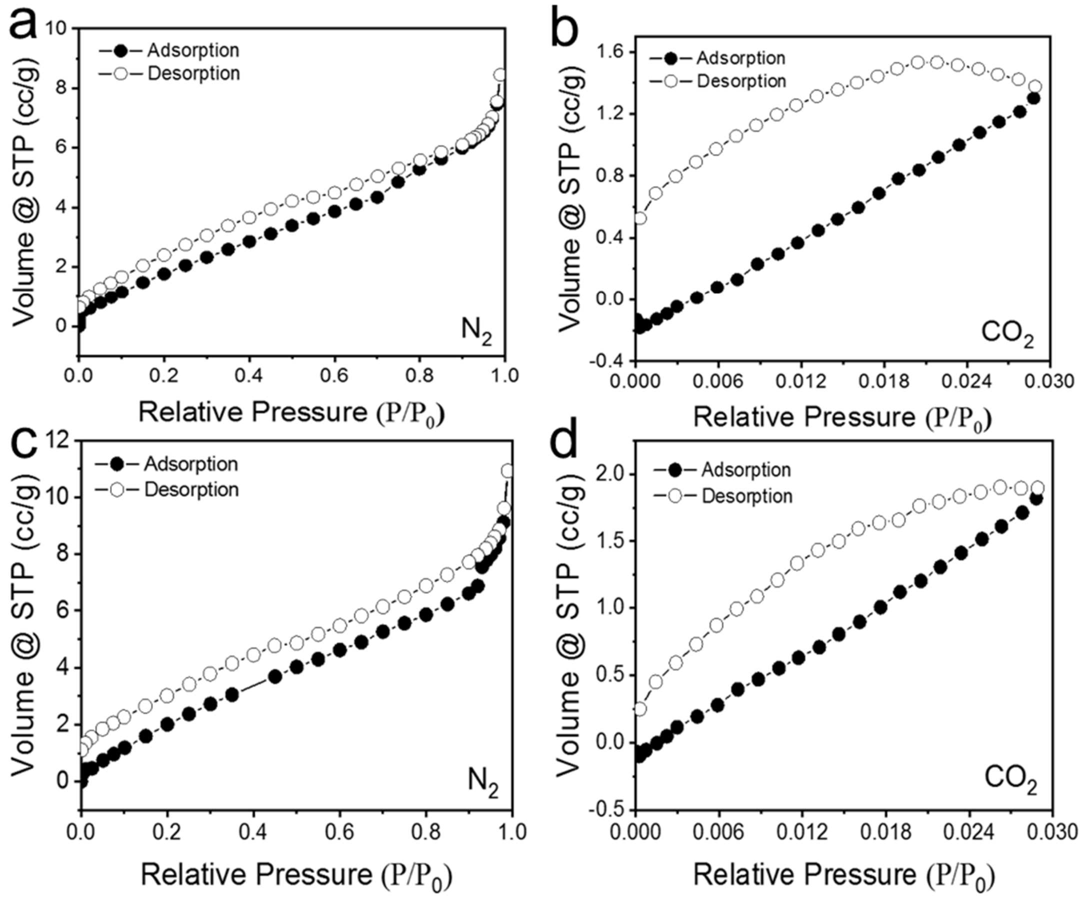

Supplementary Figure 26. Adsorption and desorption isotherms of EuTTA-300 for (a) N_2 at 77 K, and (b) CO_2 at 273 K and EuTTA-350 for (c) N_2 at 77 K, and (d) CO_2 at 273 K.

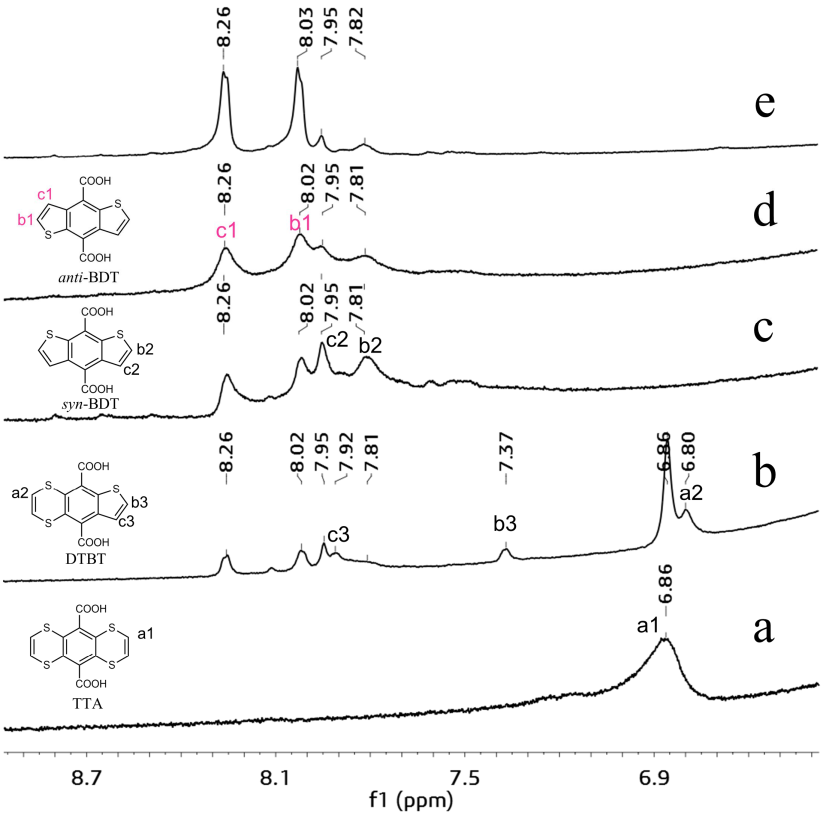

Supplementary Figure 27. Solution ^{1}H NMR spectra of: (a) as-made EuTTA crystals dissolved in HF/DMSO-d_6; (b) EuTTA-230 crystals dissolved in HF/DMSO-d_6; (c) the filtrate of a stirred mixture of EuTTA-350 crystals and HF/DMSO-d_6. (d) sample from (c) was added *anti*-BDT (2.0 mg), (e) sample from (c) was added *anti*-BDT (6.0 mg). The peaks for *anti*-BDT are consistent with the reported data, while the peaks assignments of *syn*-BDT and DTBT are tentative. The spectrum was collected on a 400 MHz Bruker superconducting magnet high-field NMR spectrometer at 298 K.

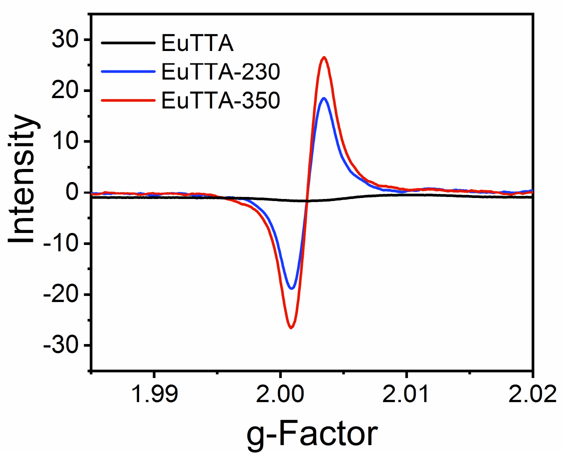

Supplementary Figure 28. Solid-state electron paramagnetic resonance (EPR) spectra of EuTTA, EuTTA-230 and EuTTA-350 powders.

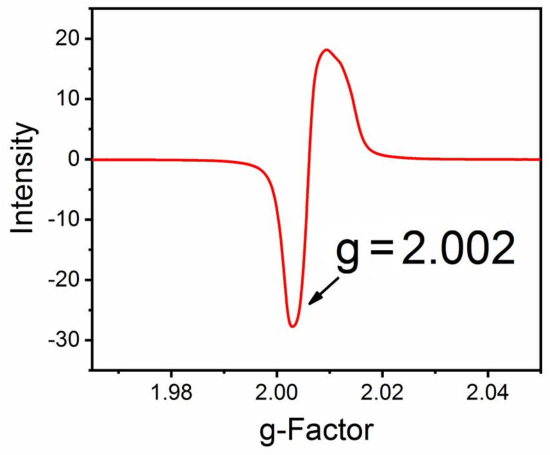

Supplementary Figure 29. Solid-state electron paramagnetic resonance (EPR) spectra of the organic components extracted from EuTTA-350 (The soluble fraction of the EuTTA-350 crystals were first extracted into DMSO/HCl, and then precipitated out by adding water; see below for the detailed procedure).

EPR sample preparation. Crystals of EuTTA-350 (20 mg) were charged into a 10-mL vial followed by addition of 1.0 mL of DMSO and 2 drops of hydrochloric acid (36%). The mixture was then stirred and heated to 60 °C for 1 hour, resulting in a dark brown solution with a little black solid remaining undissolved (<10% of the crystals used; by volume). The solution was then isolated by centrifugation, and then mixed with water (8 mL) to generate a black precipitate. The precipitate was collected by centrifugation, washed with water three times and dried in vacuum to afford the black solid sample for EPR measurement (8.0 mg).

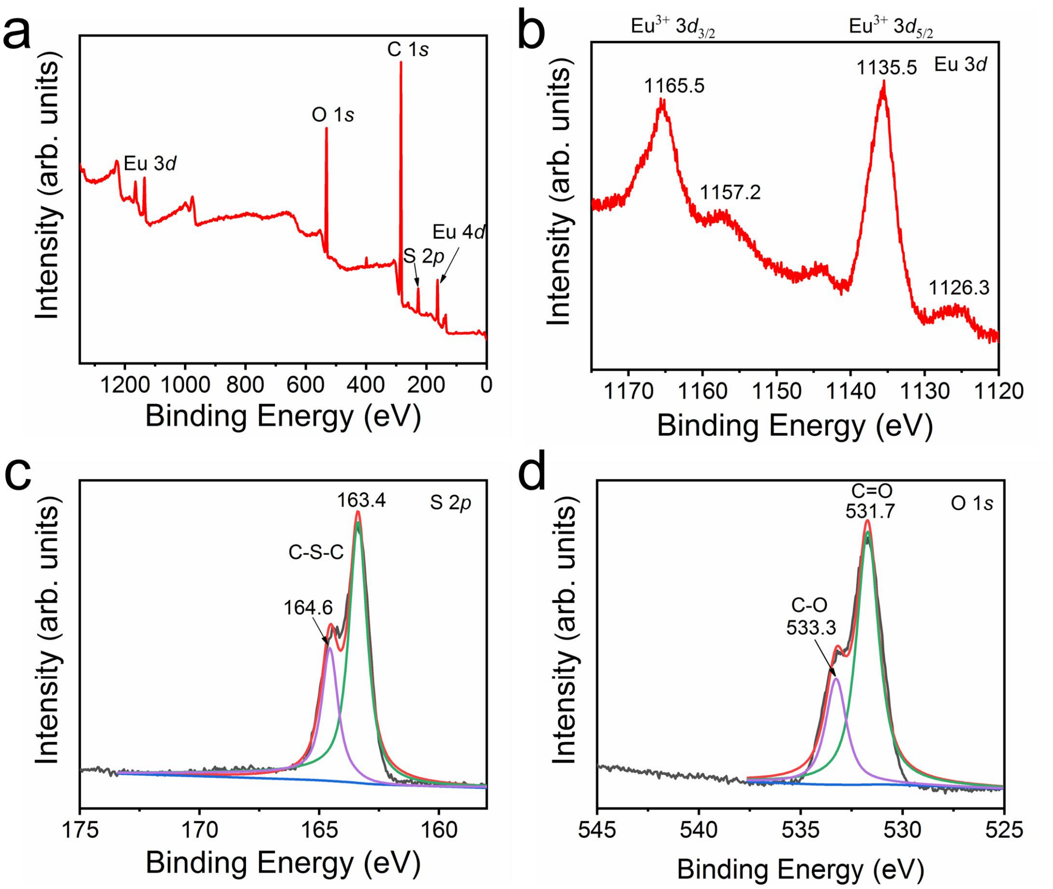

Supplementary Figure 30. A full view of the XPS spectrum (a) of a sample of EuTTA, and the close-ups for Eu 3*d* peaks (b), S 2*p* peaks (c) and O 1*s* peaks (d).

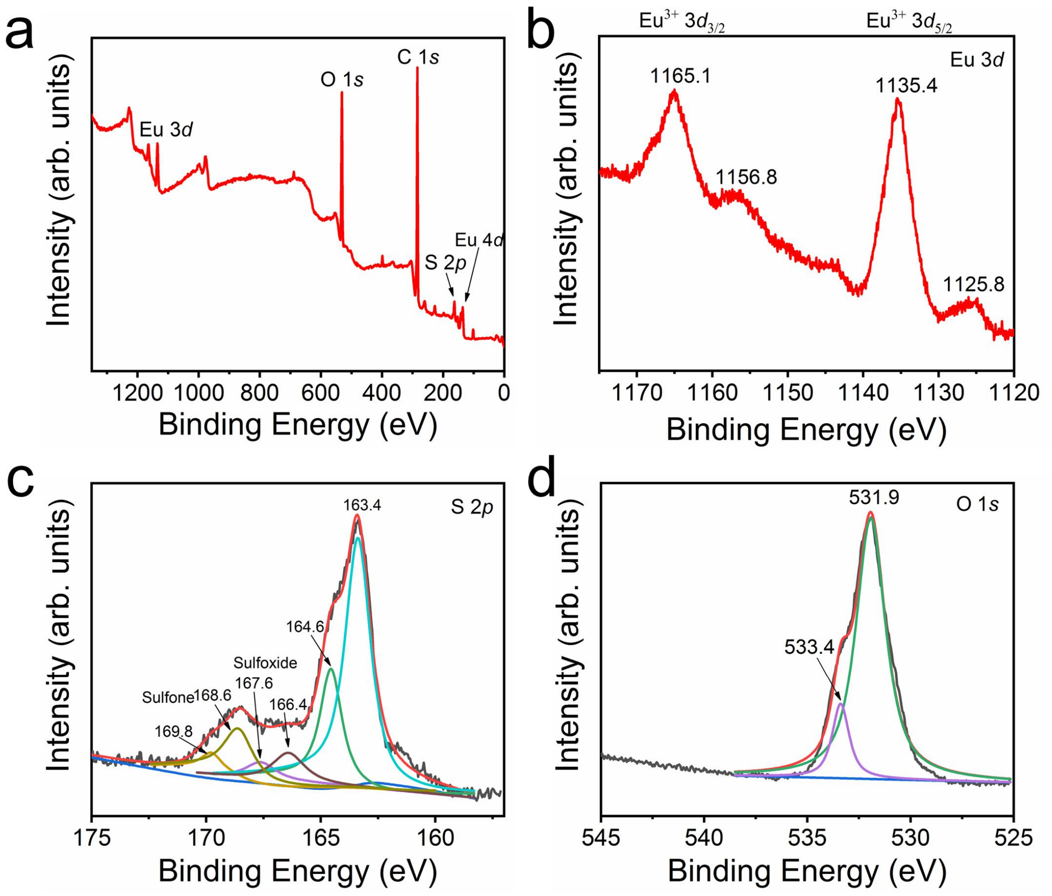

Supplementary Figure 31. A full view of the XPS spectrum (a) of a sample of EuTTA-350, and the close-ups for Eu 3*d* peaks (b), S 2*p* peaks (c) and O 1*s* peaks (d).

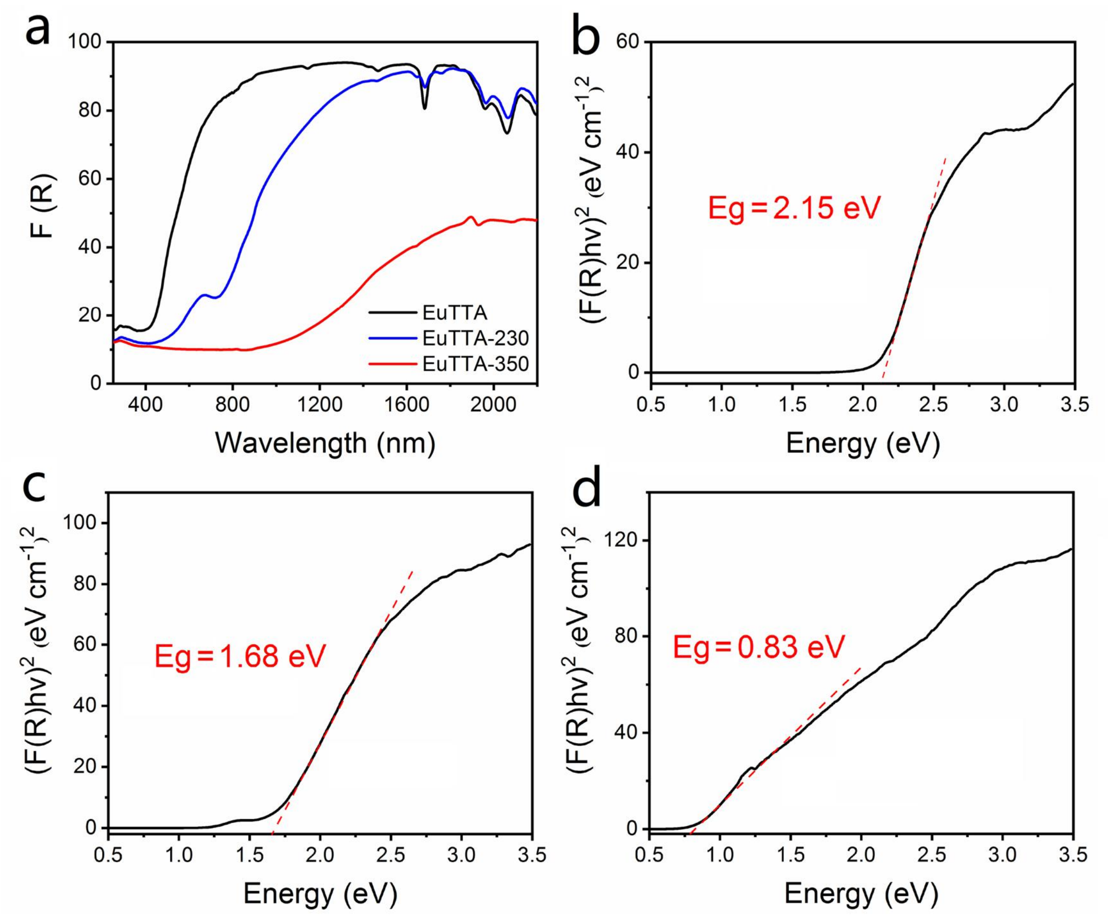

Supplementary Figure 32. (a) Normalized diffuse reflectance spectra of EuTTA, EuTTA-230 and EuTTA-350 powders; Kubelka-Munk plots of EuTTA (b), EuTTA-230 (c) and EuTTA-350 (d). Spectra are reported as the Kubelka-Munk transform, where $F(R) = (1-R)^2/2R$ (R is the diffuse reflectance of the sample as compared to $BaSO_4$).

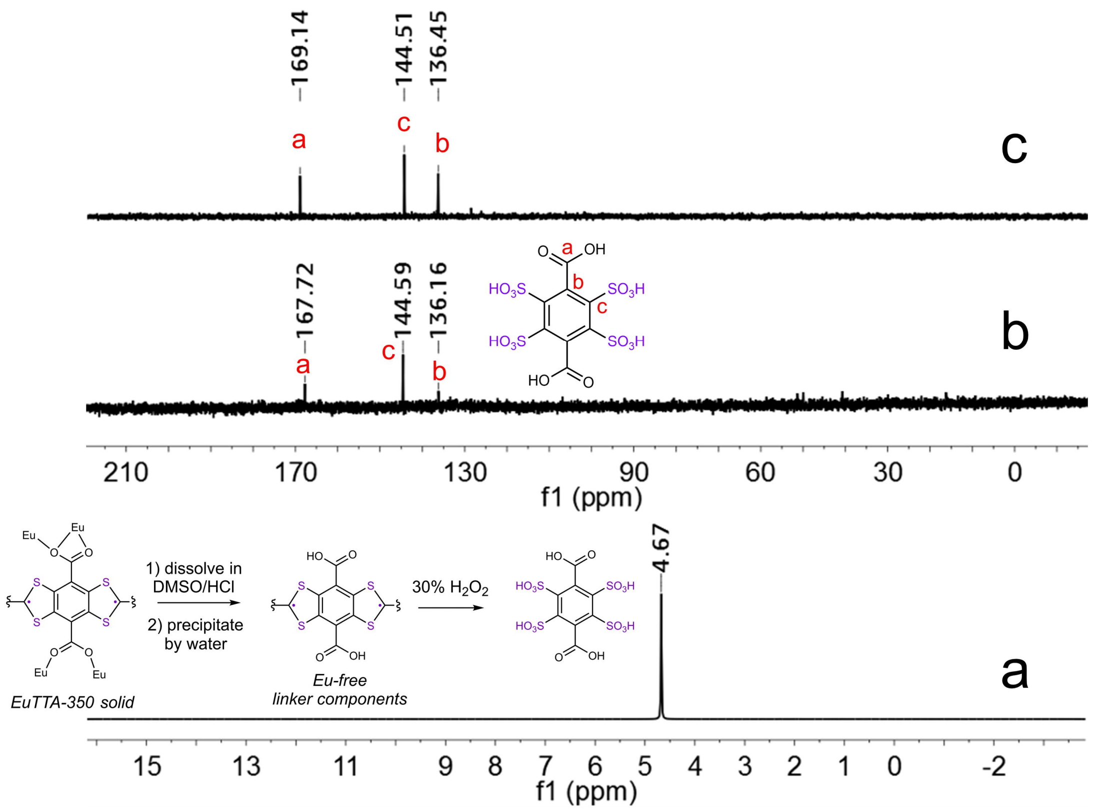

Supplementary Figure 33. Evidence for the bis(dithiole) heterocycles in EuTTA-350: The ^{1}H and ^{13}C NMR spectra (Panels a and b, respectively) of the H_2O_2-oxidized soluble components of the EuTTA-350 crystal. For comparison, the reported ^{13}C NMR spectrum of the tetrasulfonic molecule is shown in Panel c.[1] The soluble fraction of the EuTTA-350 crystals were first extracted into DMSO/HCl, and then precipitated out by adding water. The black precipitate was treated with 30% H_2O_2 to give a red-brown solution (with some red solid remaining); the solution was then evaporated, and the solid residue dissolved in D_2O for NMR measurement. The spectrum was collected on a 400 MHz Bruker superconducting magnet high-field NMR spectrometer at 298 K.

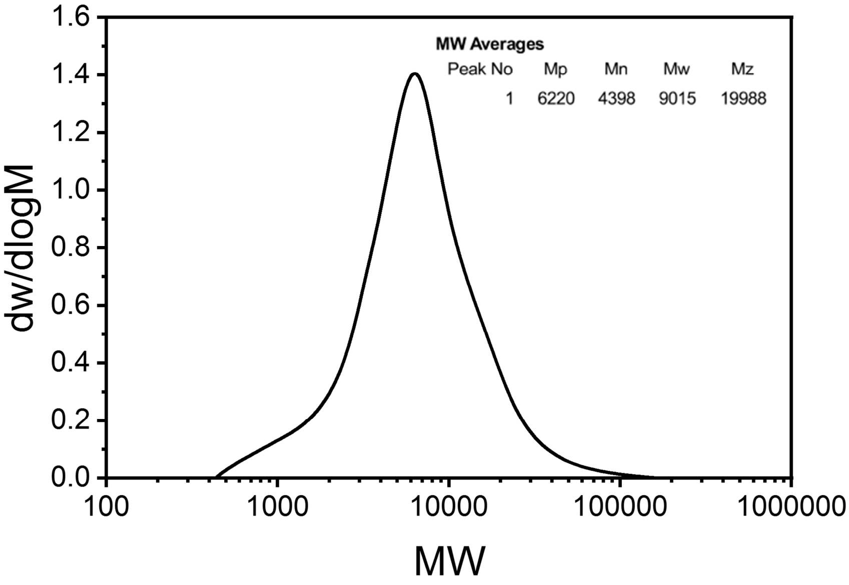

Supplementary Figure 34 Molecular weight distribution by GPC of EuTTA-350 after dissolution in DMSO/HF.

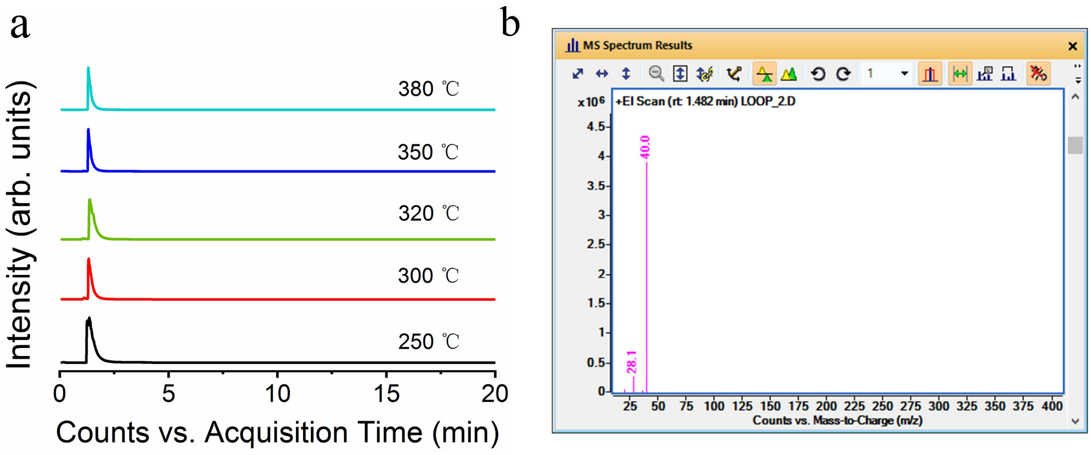

Supplementary Figure 35. Thermogravimetry, gas chromatography, mass spectrometry (TG-GC-MS): a) GC/MS chromatograms of evolved gas collected at different temperature during the thermogravimetric analysis for activated EuTTA; b) Mass spectrum of compounds detected.

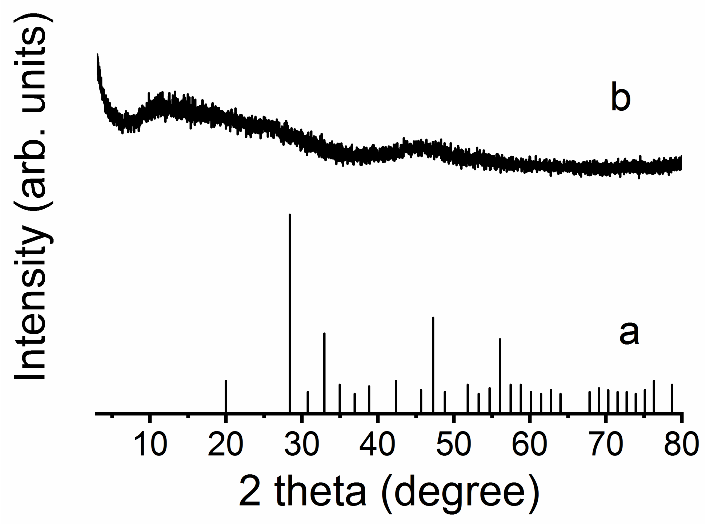

Supplementary Figure 36. PXRD patterns of (a) standard data (JCPDS 34-392) indexing the cubic Eu_2O_3 phase and (b) the insoluble black solid after dissolving the bulk of EuTTA-350 by HF/DMSO-d_6.

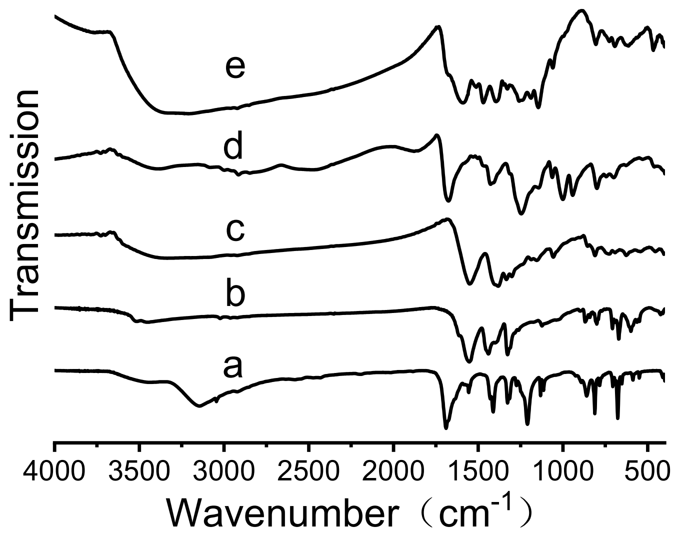

Supplementary Figure 37. The IR spectra of a) H_2TTA; b) an as-made sample of EuTTA; (c) EuTTA-350 (d) the organic components extracted from EuTTA-350 (The soluble fraction of the EuTTA-350 crystals were first extracted into DMSO/HF, and then precipitated out by adding water); (e) Insoluble residue of EuTTA-350 in DMSO/HF.

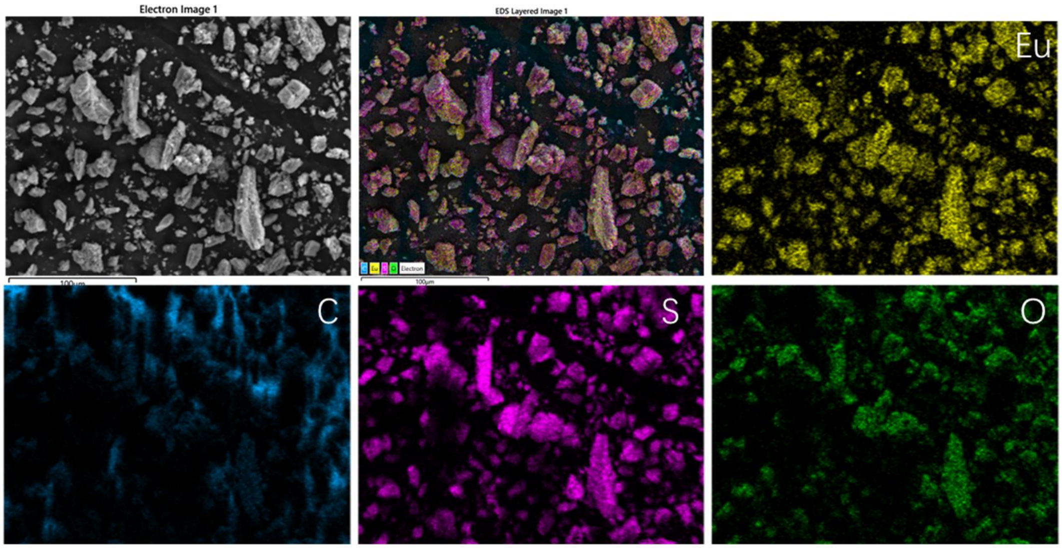

Supplementary Figure 38. An elemental mapping of insoluble residue of EuTTA-350 in DMSO/HF.

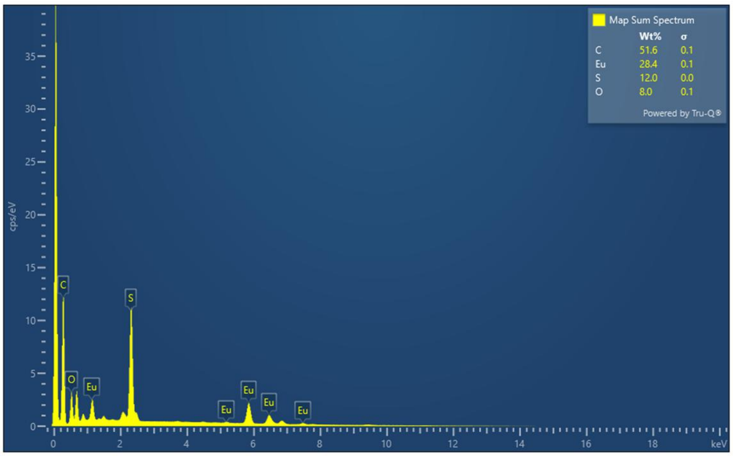

Supplementary Figure 39. EDS elemental analysis results of insoluble residue of EuTTA-350 in DMSO/HF.

Summary of the above characterization of the insoluble residue: The PXRD indicates the residue to be amorphous, and it features IR peaks indicative of organic components, which is confirmed by the substantial C, S and O elements revealed by EDS. The EDS also indicates a significant portion of Eu. The insoluble residue likely contains more extensively crosslinked organic molecules further integrated with the Eu ions into an insoluble 2D or 3D grid (in contrast to the more soluble oligomer/polymer species extracted into the solutions).

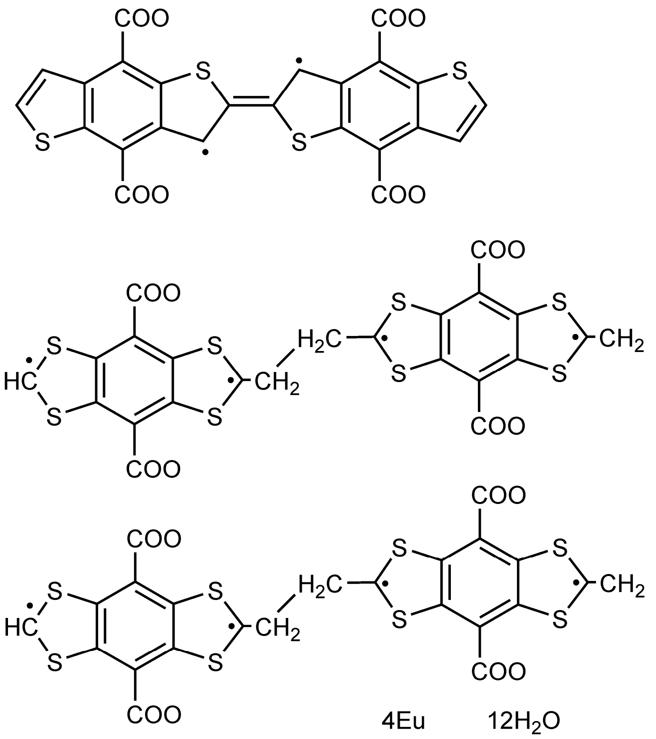

Supplementary Figure 40. A ChemDraw scheme for illustrating the crosslinking across the linker species in the 350 °C-treated sample EuTTA-350. The scheme has the formula $Eu_4(C_{24}H_6O_8S_4)_1(C_{23}H_6O_8S_8)_2(H_2O)_{14}$, and a calculated elemental profile [C (30.64 %), H (1.69 %), S (23.37 %)] consistent with the elemental analysis results [C (30.71 %), H (1.85 %), S (25.02 %)].

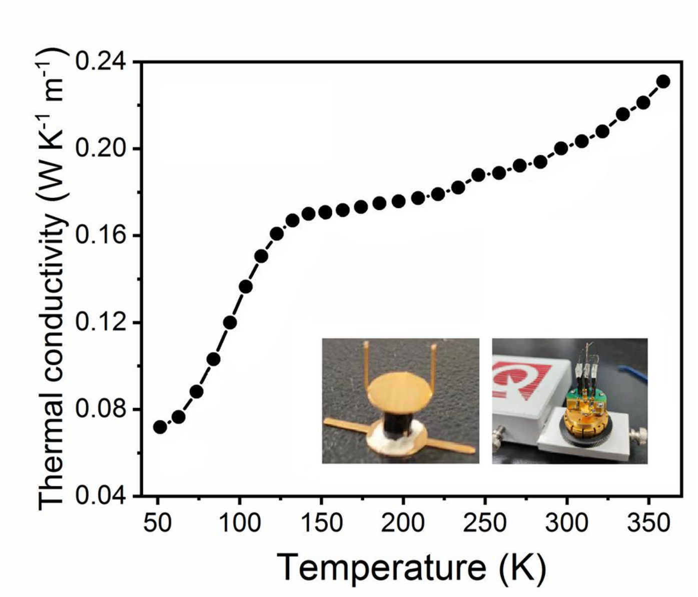

Supplementary Figure 41. Variation of the thermal conductivity of EuTTA-350 with respect to the temperature (from 50-350 K). The thermal conductivity is measured in a Physical Property Measurement System, PPMS, using the two-probe lead configuration with disk-shaped copper leads.

The thermal conductivity of EuTTA-350 at room temperature was measured by using a Physical Property Measurement System, PPMS, and found to be about 0.2 W K^{-1} m^{-1}. Since EuTTA-350 is electrically insulating, the contribution to the thermal conductivity by phonons is larger than that by electrons.[2] As the temperature decreases, the thermal conductivity becomes lower probably stemming from phonon scattering.[3] These results suggest that EuTTA-350 has good thermal insulation performance.

Computational details

Density Functional Theory (DFT) calculation was used to study the nature of the biradical on contracted TTA linkers resulting from thermal annealing. Geometry optimizations and frequency calculation[4] were performed using ORCA 4.2.1[5] with B3LYP[6] functional for density-fitting approximation and def2-TZVP[7,8] basis set. The multiplicity of the structure was set to be triplet consistent with biradical state. In the geometry optimization, the default tight convergence in the SCF cycle was adopted without any orbital symmetry constraints. The optimized structure was confirmed to be energetically at its local minimum without imaginary/negative

frequencies before further calculation. Mulliken population analysis spin natural orbital (SNO) by Becke method[9] and spin density analysis were performed using Multiwfn 3.7 software package[10]. Spin density, spin population and SNOs were plotted with VMD 1.9.3[11].

Supplementary Table 1. Cartesian coordinates, Mulliken charge and spin density of bis(dithiole).

	Atomic coordinates (Cartesian in *x*, *y*, *z*; in atomic unit)			Mulliken charge	Spin density
C1	-2.13133717661087	3.49031592100323	0.09893390882327	0.14806	0.11068
C2	-3.23779036255765	2.61670209788568	0.13609218282680	-0.14443	0.04401
C3	-3.07285779454594	1.23601040473645	0.06543686153760	-0.17803	0.01105
C4	-1.77722575773462	0.67197842189987	-0.01853299939607	0.16493	0.09714
C5	-0.66015549495281	1.53298321125832	0.02623760216623	-0.17463	-0.02242
C6	-0.84160485332443	2.92986488411131	0.11414611335146	-0.17668	-0.02256
S7	0.61623859885813	3.90128839155718	0.23434953319586	0.37092	0.09288
S8	1.01257145695430	1.00395575944305	-0.03117338420212	0.34522	0.07665
S9	-4.89781572335510	3.17531003549037	0.37699996580293	0.19753	0.12559
S10	-4.54370055316676	0.26408402872392	0.09221151816493	0.32892	0.15291
C11	-5.61151335235238	1.62904875690986	0.07520825698302	-0.43971	0.70683
C12	1.68608322102686	2.56967960040840	0.09853995459052	-0.45113	0.62534
C13	-2.22559185799879	4.97038766858631	0.00939688267808	0.49071	0.00677
C14	-1.67933654607292	0.78669504978596	-0.10906108511327	0.45985	0.00910
O15	-1.30446642999209	5.68046142186256	0.33830987650403	-0.47491	0.01219
O16	-2.63058572079130	1.53899723973405	-0.08148687987845	-0.49744	0.02694
O17	-3.33513930356034	5.52939919909355	-0.51422546953563	-0.37588	0.00739
O18	-0.41241756275804	1.24729413013699	-0.22688495000555	-0.42237	0.00000
H19	-3.99276181338131	4.86256308258731	-0.78115800288038	0.26268	0.00971
H20	-0.46600808170852	2.21505902432148	-0.26914764301027	0.29160	-0.00049
H21	-6.65239934500073	1.48276847695673	0.31838483295575	0.12831	-0.03260
H22	2.75185445302536	2.71622408146432	0.04743292444127	0.14649	-0.03713

Orbital component analysis (Becke method)

Supplementary Table 2. Spin natural orbital (SNO) contribution of two spins on bis(dithiole).

Atom	SNO1 Contribution (%)	SNO2 Contribution (%)
C1	4.998	1.540
C2	3.070	0.888
C3	2.678	0.710
C4	4.339	1.412
C5	0.804	0.958
C6	0.813	0.923
S7	16.078	0.266
S8	15.077	0.281

S9	1.138	18.679
S10	1.214	19.887
C11	0.049	49.909
C12	40.567	0.060
C13	1.758	0.184
C14	1.624	0.571
O15	1.198	0.176
O16	1.702	0.430
O17	0.681	0.375
O18	0.405	0.217
H19	0.037	0.440
H20	0.007	0.006
H21	0.002	2.086
H22	1.760	0.001

The following optimized geometry was displayed in Cartesian coordinate system. E° represents the electronic energy of the optimized structure, and G° represents the total thermal energy in Hartree unit.

TTA [E° = -2276.89878759; G° = -2276.76121305]

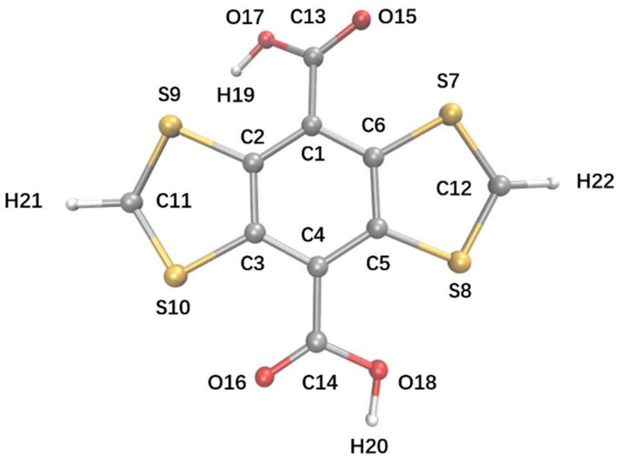

Charge = 1; Multiplicity = 3; Total spin electrons = 2

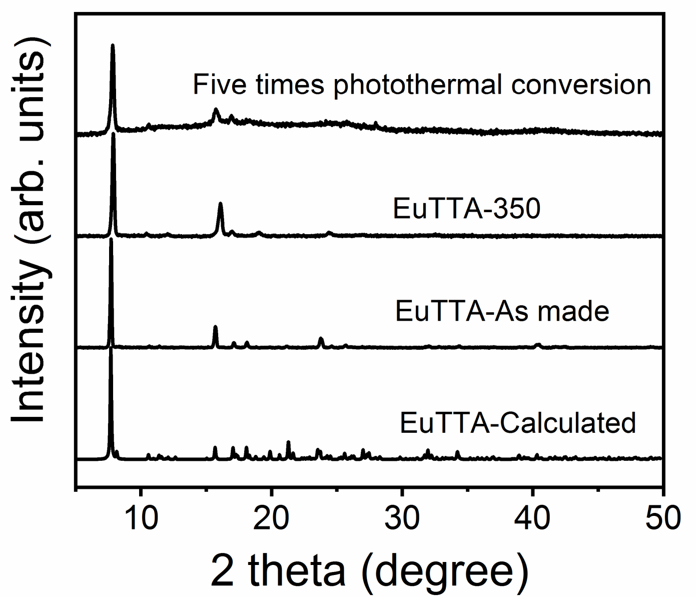

Supplementary Figure 42. PXRD patterns of EuTTA-350 after 5 photothermal conversion cycles.

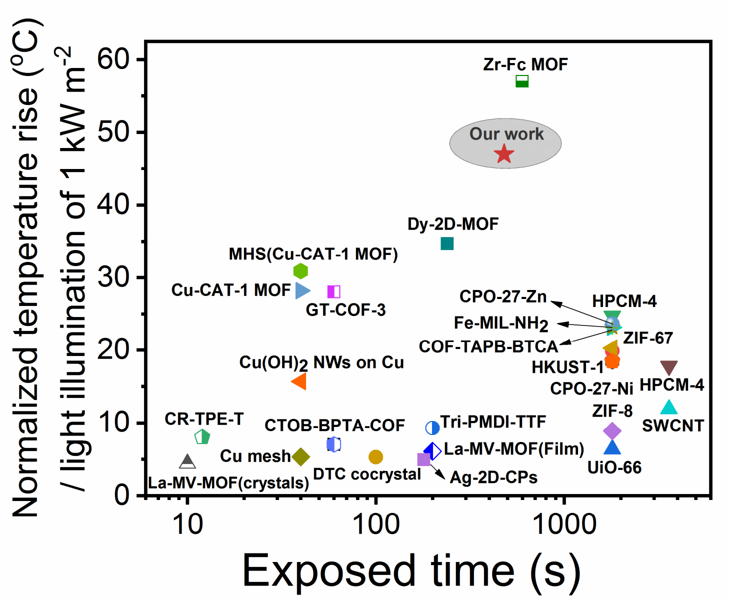

Supplementary Figure 43. A plot of the maximum temperature rises and the corresponding time (in seconds) entailed for various photothermal materials (normalized by light illumination of 1 kW m^{-2}). NOTE: The values of Zr-Fc-MOF measured in our hands are lower than the reported numbers shown in this plot, *e.g.*, rising by only 34.4 °C; and we are still trying to find the cause.

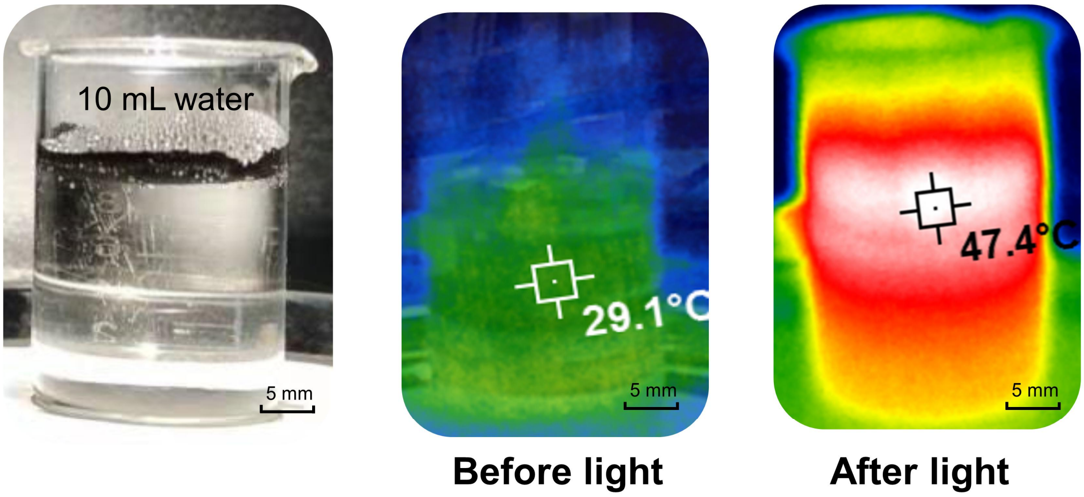

Supplementary Figure 44. The optical photographs and the corresponding IR photographs of an EuTTA-350 (50 mg) powder floating on the water surface: the IR pictures were taken by an IR camera before and after irradiated for 1 hour by light illumination of 1 kW m^{-2} (10 mL beaker, inner diameter of 22 mm).

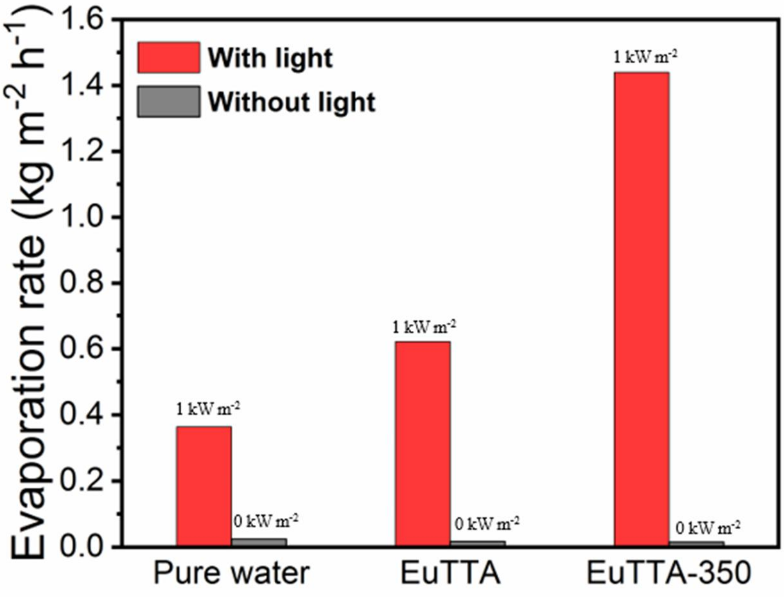

Supplementary Figure 45. A bar chart for water evaporation rates of pure water, water with EuTTA (50 mg) and water with EuTTA-350 (50 mg) under xenon lamp irradiation (red bars) and without xenon lamp irradiation (grey bars).

Calculation of the efficiency for solar to vapor generation. The conversion efficiency η of solar energy in photothermal assisted water evaporation was calculated as the following formula.

$$\eta = \frac{\dot{m} h_{LV}}{C_{opt} P_0} \tag{1}$$

Where $\dot{m}$ refers to the mass flux of water (evaporation rate, see **Supplementary Figure 45**), h_{LV} refers to the total liquid-vapor phase transition enthalpy [*i.e.*, sensible heat and vaporization enthalpy (h_{LV} = $Q + \Delta h_{vap}$)], and Q is the energy used to transform the system from the initial temperature T_0 is heated to the final temperature T, Δh_{vap} is the latent heat of vaporization of water; P_0 is the nominal solar radiation value of 1 kW m^{-2}; and C_{opt} represents the optical concentration. The schematic diagram of the vaporization enthalpy of steam is as follows:

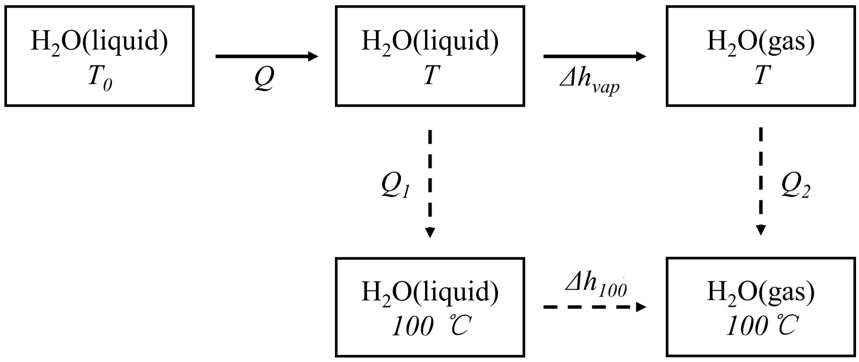

$$Q = C_{liquid} \times (T - T_0) \tag{2}$$

$$\Delta h_{vap} = Q_1 + \Delta h_{100} + Q_2 \tag{3}$$

$$Q_1 = C_{liquid} \times (100 - T) \tag{4}$$

$$Q_2 = C_{vapor} \times (T - 100) \tag{5}$$

In this work, C_{liquid}, the specific heat capacity of liquid water is a constant of 4.18 J g^{-1} °C^{-1} C_{vapor}, the specific heat capacity of water vaper is a constant of 1.865 J g^{-1} °C^{-1}. Δh_{100} is the latent heat of vaporization of water at 100 °C, taken to be 2260 kJ kg^{-1}.

For example, the surface temperature of EuTTA-350 (50 mg) was 47.4 °C during the evaporation process (**Supplementary Figure 44**), therefore T is 47.4 °C. According to the above formulas,

Q = 4.18 × (47.4 - 25) = 93.632 kJ kg^{-1}

Δh_{vap} = 4.18 × (100 - 47.5) + 2260 + 1.865 × (47.5 - 100) = 2381.769 kJ kg^{-1}

$h_{LV} = Q + \Delta h_{vap} = 93.632 + 2381.769 = 2475.401$ kJ kg^{-1}

$\dot{m} = 1.440 - 0.016 = 1.424$ kg m^{-2} h^{-1}

$P_0 = 1$ kW m^{-2}

$C_{opt} = 1$

$$\eta = \dot{m}h_{LV}/C_{opt}P_0 = \frac{1.424 \times 2475.401}{3600} = 97.9\%$$

As a result, evaporation efficiency η = 97.9% when the latent heat of water vaporization at 47.4 °C (2392 kJ kg^{-1}) is used in calculation.

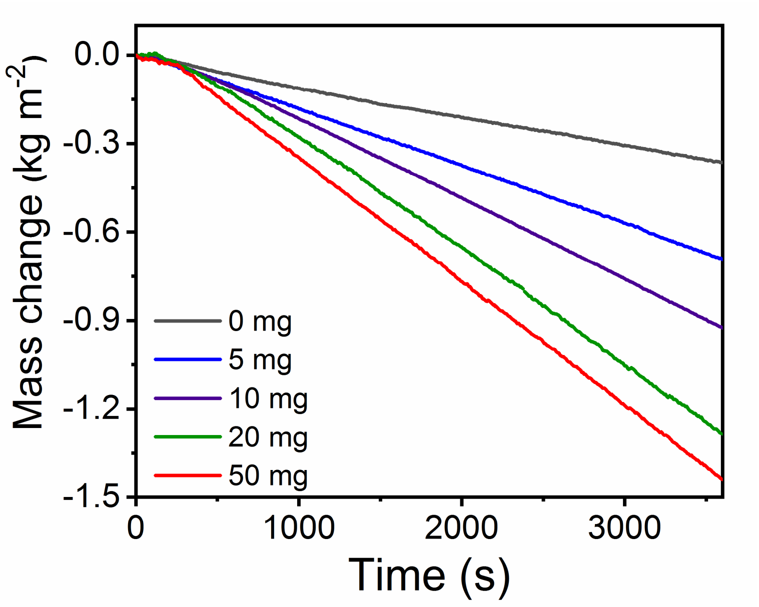

Supplementary Figure 46. A plot of mass loss of water against time, with the mass loss being enabled by 0, 5, 10, 20 and 50 mg of EuTTA-350 powders under 1-sun (1 kW m^{-2}).

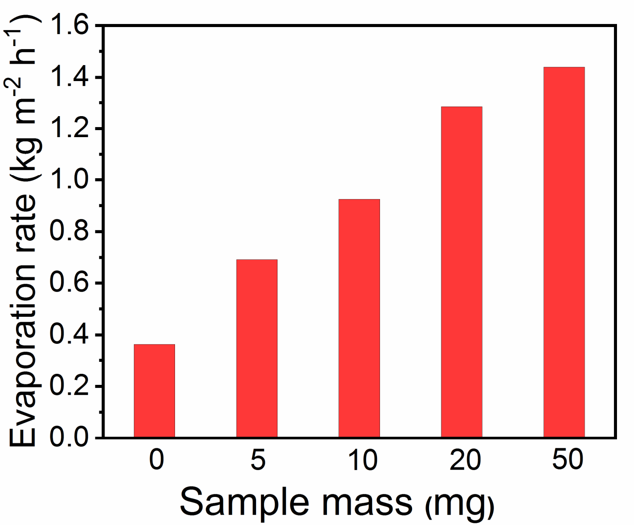

Supplementary Figure 47. Water evaporation rates as enabled by 0, 5, 10, 20 and 50 mg of EuTTA-350 powders under 1-sun (1 kW m^{-2}).

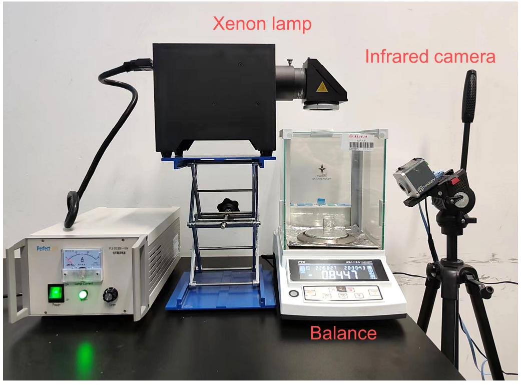

Supplementary Figure 48. Photographs for the device for recording the water mass change under solar irradiation. Under 1-sun (1 kW m^{-2}) exposure in air, this simulation device was set up to monitor the changes in water weight over time (10 mL beaker, inner diameter of 22 mm).

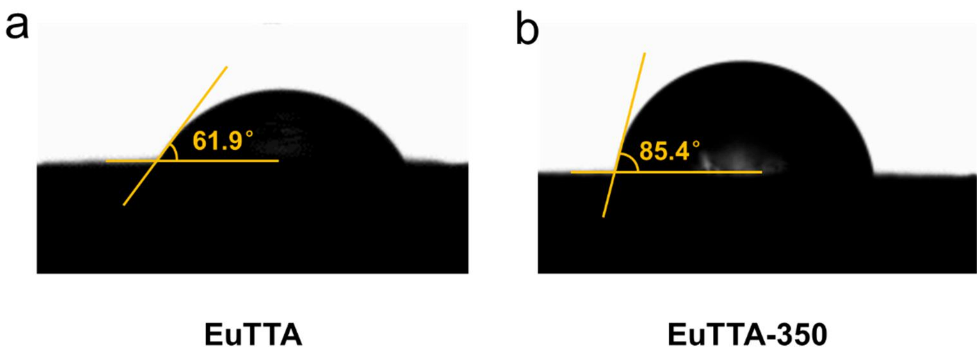

Supplementary Figure 49. Photographs for the water contact angle measurement of a pressed pellet of (a) a sample of EuTTA (contact angle: 61.9°); (b) a sample of EuTTA-350 (contact angle: 85.4°).

Supplementary Table 3. Performance metrics of reported materials for solar-driving water evaporation. (1 kW m^{-2})

No	System	Rate (kg m^{-2} h^{-1})	Solar-to-vapor efficiency (%)	References
1	Carbon based on vapor generators	1.62	100	Ref.[12]
2	Loofah and Co-MOF	1.93	98.5	Ref.[13]
3	EuTTA-350	1.44	97.9	Our work
4	Cu-CAT-1	1.50	97.6	Ref.[14]
5	Zr–Fc MOF/SWCNT/gelatin (ZSG) membrane	1.53	95.6	Ref.[15]
6	TPAD-COF	1.42	94 .0	Ref.[16]
7	HBG	1.36	93.7	Ref.[17]
8	Hierarchical graphene foam	1.40	93.4	Ref.[18]
9	Co-CNS/M foam	1.393	93.4	Ref.[19]
12	SWCNT/Gelatin thin membrane(HKUST-1)	1.39	92.0	Ref.[20]
13	Ni-MOFs	2.07	91.5	Ref.[21]
14	HKUST-1/SWCNT Hybrid Membranes	1.38	90.8	Ref.[22]
15	GT-COF-3	1.314	90.7	Ref.[23]
16	GDPA-QCN cellulose paper	1.30	90.4	Ref.[24]
17	O-KFs-MXene aerogels	1.47	90.4	Ref.[25]
18	PU+TPA-TPA-O_6	1.293	89.41	Ref.[26]
19	GCMP-2	1.41	89.3	Ref.[27]
20	MOF-801@CL	1.42	88.9	Ref.[28]
21	GCMP-1	1.36	87.9	Ref.[27]

22	CR-TPE-T	1.272	87.2	Ref.[29]
23	BHMS	1.39	84.7	Ref.[30]
24	MDPC/SS mesh	1.222	84.3	Ref.[31]
25	HKUST-1/CNF	1.33	84.3	Ref.[32]

Supplementary Table 4. Crystal Data and Structure Refinement Parameters for EuTTA and EuTTA-230.

**Compound**	EuTTA	EuTTA-230
**Chemical formula**	$C_{144}H_{48}Eu_8O_{52}S_{48}$	$C_{72}H_{28}Eu_4O_{26}S_{20}$
**Formula weight**	5364.38	2557.98
**Space group**	C2/c	C2/c
**a (Å)**	16.5676(15)	16.385(3)
**b (Å)**	16.3937(14)	16.484(3)
**c (Å)**	16.2839(14)	16.295(3)
**α (°)**	90	90
**B (°)**	113.838(3)	116.11(3)
**γ (°)**	90	90
**V ($Å^3$)**	4045.5(6)	3952.0(15)
Z	4	4
**Dc ($g.cm^{-3}$)**	2.202	2.150
**F (000)**	2600.0	2480
**GOF**	1.083	1.083
**R_1^a[I>2σ (I)]**	0.0841	0.1106
**wR_2^b (all data)**	0.2168	0.2761

^{a} $R_1 = \sum||Fo| - |Fc|| / \sum(|Fo|)$

^{b} $wR_2 = \{\sum[w(Fo^2 - Fc^2)^2] / \sum[w(Fo^2)^2]\}^{1/2}$

Supplementary References

1. He, Y. *et al.* Dense Dithiolene Units on Metal–Organic Frameworks for Mercury Removal and Superprotonic Conduction. *ACS Appl. Mater. Interfaces* ***14,*** 1070–1076 (2022).
2. Sun, L. *et al.* A Microporous and Naturally Nanostructured Thermoelectric Metal-Organic Framework with Ultralow Thermal Conductivity. *Joule* **1**, 168-177 (2017).
3. Wu, S., Yan, T., Kuai, Z. & Pan, W. Thermal conductivity enhancement on phase change materials for thermal energy storage: A review. *Energy Stor. Mater.* **25**, 251-295 (2020).
4. Grimme, S. Supramolecular Binding Thermodynamics by Dispersion-Corrected Density Functional Theory. *Chem. Eur. J.* **18**, 9955-9964 (2012).
5. Neese, F. Software update: the ORCA program system, version 4.0. *WIREs Comput Mol Sci.* **8**, e1327 (2018).
6. Dorn, H. C., Yannoni, C. S., Limbach, H.-H. & Vogel, E. Evidence for a Nonclassical Structure of a 1,6-Methano[10]annulene: A Cryogenic 13C CPMAS NMR Study of the 11,11-Dimethyl Derivative. *J. Phys. Chem.* **98**, 11628-11629 (1994).
7. Weigend, F. & Ahlrichs, R. Balanced basis sets of split valence, triple zeta valence and quadruple zeta valence quality for H to Rn: Design and assessment of accuracy. *Phys. Chem. Chem. Phys.* **7**, 3297-3305 (2005).
8. Weigend, F. Accurate Coulomb-fitting basis sets for H to Rn. *Phys. Chem. Chem. Phys.* **8**, 1057-1065 (2006).
9. Tian, L. U. & Feiwu, C. Calculation of molecular orbital composition. *Acta Chim. Sinica* **69**, 2393-2406 (2011).
10. Lu, T. & Chen, F. Multiwfn: A multifunctional wavefunction analyzer. *J Comput Chem* **33**, 580-592 (2012).
11. Humphrey, W., Dalke, A. & Schulten, K. VMD: Visual molecular dynamics. *J. Mol. Graph.* **14**, 33-38 (1996).
12. Li, X. *et al.* Enhancement of Interfacial Solar Vapor Generation by Environmental Energy. *Joule* **2**, 1331-1338 (2018).
13. Wang, H. *et al.* Biomimetic Ultra-Black Sponge Derived from Loofah and Co-MOF for Long-Term Solar-Powered Vapor Generation and Desalination. *Solar RRL* **5**, 2000817 (2021).
14. Ma, Q. *et al.* MOF-Based Hierarchical Structures for Solar-Thermal Clean Water Production. *Adv. Mater.* **31**, 1808249, (2019).
15. Ma, X. *et al.* A photothermal and Fenton active MOF-based membrane for high-efficiency solar water evaporation and clean water production. *J. Mater. Chem. A* **8**, 22728-22735 (2020).
16. Yan, X. *et al.* Superhydrophilic 2D Covalent Organic Frameworks as Broadband Absorbers for Efficient Solar Steam Generation. *Angew. Chem. Int. Ed.* **61**, e202201900 (2022).
17. Ai, Z. *et al.* Self-assembly hierarchical binary gel based on MXene and montmorillonite nanosheets for efficient and stable solar steam generation. *Journal of Cleaner Production* **357**, 132000 (2022).
18. Ren, H. *et al.* Hierarchical Graphene Foam for Efficient Omnidirectional Solar–Thermal Energy Conversion. *Adv. Mater.* **29**, 1702590 (2017).
19. Fan, X. *et al.* A MXene-Based Hierarchical Design Enabling Highly Efficient and Stable Solar-Water Desalination with Good Salt Resistance. *Adv. Funct. Mater.* **30**, 2007110 (2020).
20. Ma, X. *et al.* A robust asymmetric porous SWCNT/Gelatin thin membrane with salt-resistant for efficient solar vapor generation. *Appl. Mater. Today* **18**, 100459 (2020).

21. He, P. *et al.* Controllable synthesis of sea urchin-like carbon from metal-organic frameworks for advanced solar vapor generators. *Chem. Eng. J.* **423**, 130268 (2021).
22. Ma, X. *et al.* Hierarchical Porous SWCNT Stringed Carbon Polyhedrons and PSS Threaded MOF Bilayer Membrane for Efficient Solar Vapor Generation. *Small* **15**, 1900354 (2019).
23. Tang, X. *et al.* Design of Photothermal Covalent Organic Frameworks by Radical Immobilization. *CCS Chemistry* **0**, (2021).
24. Liu, J. *et al.* Donor-Acceptor Molecule Based High-Performance Photothermal Organic Material for Efficient Water Purification and Electricity Generation. *Angew. Chem. Int. Ed.* **61**, e202117087 (2022).
25. Chen, L. *et al.* MXene-doped kapok fiber aerogels with oleophobicity for efficient interfacial solar steam generation. *J. Colloid Interface Sci.* **626**, 35-46 (2022).
26. Wang, Z., Zhou, J., Zhang, Y., Zhu, W. & Li, Y. Accessing Highly Efficient Photothermal Conversion with Stable Open-Shell Aromatic Nitric Acid Radicals. *Angew. Chem. Int. Ed.* **61**, e202113653 (2022).
27. Ma, Y. *et al.* Highly efficient solar photothermal conversion of graphene-coated conjugated microporous polymers hollow spheres. *J. Colloid Interface Sci.* **623**, 856-869 (2022).
28. Guo, M.-X., Wu, J.-B., Zhao, H.-Y., Li, F.-H. & Min, F.-Q. Carbonized loofah and MOF-801 of synergistic effect for efficient solar steam generation. *Int. J. Energy Res.* **45**, 10599-10608 (2021).
29. Chen, G. *et al.* Biradical-Featured Stable Organic-Small-Molecule Photothermal Materials for Highly Efficient Solar-Driven Water Evaporation. *Adv. Mater.* **32**, 1908537, (2020).
30. Cui, W.-R., Zhang, C.-R., Liang, R.-P., Liu, J. & Qiu, J.-D. Covalent Organic Framework Sponges for Efficient Solar Desalination and Selective Uranium Recovery. *ACS Appl. Mater. Interfaces* **13**, 31561-31568 (2021).
31. Ma, S., Qarony, W., Hossain, M. I., Yip, C. T. & Tsang, Y. H. Metal-organic framework derived porous carbon of light trapping structures for efficient solar steam generation. *Sol. Energy Mater Sol. Cells* **196**, 36-42 (2019).
32. Zhao, X., Ma, X. & Peng, X. Carbon nanofiber stringed hierarchical porous carbon polyhedrons flexible thin films for solar vapor generation. *Appl. Phys. A* **125**, 537 (2019).
